# High Resolution Treatment Effects Estimation: Uncovering Effect Heterogeneities with the Modified Causal Forest

**DOI:** 10.3390/e24081039

**Published:** 2022-07-28

**Authors:** Hugo Bodory, Hannah Busshoff, Michael Lechner

**Affiliations:** 1 Vice-President’s Board (Research & Faculty), University of St. Gallen, Dufourstrasse 50, 9000 St. Gallen, Switzerland; hugo.bodory@unisg.ch; 2 Swiss Institute for Empirical Research, University of St. Gallen, Varnbüelstrasse 14, 9000 St. Gallen, Switzerland; michael.lechner@unisg.ch

**Keywords:** econometrics software, causal machine learning, statistical learning, conditional average treatment effects, individualized treatment effects, multiple treatments, selection-on-observables, C21, C870, J68

## Abstract

There is great demand for inferring causal effect heterogeneity and for open-source statistical software, which is readily available for practitioners. The mcf package is an open-source Python package that implements Modified Causal Forest (mcf), a causal machine learner. We replicate three well-known studies in the fields of epidemiology, medicine, and labor economics to demonstrate that our mcf package produces aggregate treatment effects, which align with previous results, and in addition, provides novel insights on causal effect heterogeneity. For all resolutions of treatment effects estimation, which can be identified, the mcf package provides inference. We conclude that the mcf constitutes a practical and extensive tool for a modern causal heterogeneous effects analysis.

## 1. Introduction

Supervised machine learning algorithms, which learn a model by minimizing prediction errors, do not generalize per se to evaluate treatment effects due to the missing data problem. For each unit of observation, only one potential outcome is observed; hence, the individualized treatment effect (ITE) remains unknown. This disallows to train a model by minimizing the prediction error of the ITE. With the onset of causal machine learning in recent years, flexible methods have been developed, which integrate supervised machine learners into the classical analysis of causality. The causality literature defines the set of conditions required to identify the causal parameters of interest and deals with the missing data by imputing counterfactuals for adequate subpopulations [1], while the machine learning (ML) literature provides methods to flexibly estimate treatment effects and deal with a potentially large number of features. The causal machine learning literature has also opened the door to systematic heterogeneous treatment effects estimation. There is considerable interest in understanding heterogeneous treatment effects in various scientific fields, including business, economics, epidemiology, marketing, and medicine (as discussed in, e.g., [2]). The underlying premise is that treatment responses vary for subpopulations. Uncovering this variation informs our understanding of the distributional implications of a treatment and the underlying causal mechanisms, and potentially hints at more efficient targeting rules.

Ref. [3] structure the rich universe of causal machine learners. They distinguish between generic causal machine learners, which integrate a variety of off-the-shelf machine learning estimators, e.g., [4], and estimator-specific approaches, where a specific machine learner is adapted to the causal question, e.g., the tree-based methods [5,6,7,8].

The causal forest by [7] is most related to the mcf estimator [9]. In each tree in the causal forest, the feature space is recursively split to maximize the implied effect heterogeneity greedily. The authors of [7] showed that this is equivalent to minimizing the mean squared prediction error of treatment effects. Treatment effects are obtained as leaf-specific average differences averaged over all trees in the forest. Ref. [9] innovated the causal forest estimator [5,7] in two dimensions. First, the splitting criterion in the tree growing step is adapted to account for covariance structures in estimation errors of mean conditional outcomes and selection bias. Ref. [9] demonstrated in extensive simulations that the bias adjustment results in considerable performance improvements. Second, ref. [9] stipulated a computationally efficient outcome-weight-based approach, which facilitates an approximate inference of causal effects at all levels of resolution from estimating the modified causal forest once.

Since June 2021, an open-source Python implementation of the estimator has been made available on the Python Package Index (PyPI). The Python package provides an off-the-shelf tool for practitioners to analyze effect heterogeneity for multiple treatment models in a selection-on-observables setting. Related statistical software includes the Python package EconML [10] and the R package grf [11]. Both implement forest-based causal machine learners (orthogonal random forest, forest double machine learning estimator, forest doubly robust estimator, the generalized random forest). However, in contrast to the mcf, the cited packages do not infer causal effects at all levels of resolution in one estimation round.

We present the package and demonstrate its core functionality—inference of heterogeneous causal effects at different levels of resolution—in the replication of three well-published studies in the realm of epidemiology, medicine, and labor economics. Code and data can be accessed on GitHub [12]. We found that the mcf matches results on aggregate treatment effects estimation and provides additional insights on underlying effect heterogeneity as measured by the individualized and group average treatment effects.

We contribute to the literature in five dimensions: First, we present the open-source Python package that implements the mcf. Second, we provide novel results on causal effect heterogeneity for benchmark studies in epidemiology, medicine, and labor economics. In that scope, we demonstrate that the mcf matches previous results on aggregate treatment effects and effectively deals with binary and multi-valued treatments and arbitrary outcome and feature distributions. Third, for all resolutions of causal effect heterogeneity, which can be statistically identified, we provide inference. Fourth, we uncover relevant effect heterogeneity, which is potentially instructive for tailoring treatment assignments in constrained settings. Fifth, we provide data, data documentation, and code to replicate our results.

The remainder of this paper proceeds as follows. In Section 2, we delineate identification, the estimands of interest, estimation, and the package’s infrastructure. For a detailed discussion of the methodology, refer to [9]. Section 3 presents the results of our replications. Finally, Section 4 concludes.

## 2. Framework

The mcf is a tree-based causal machine learner that produces valid causal estimates in the selection-on-observables setting. To set the scene, we detail the identification setting, define the causal parameters of interest at different levels of resolution, and outline the core ideas of the mcf and the package’s infrastructure. For details of the algorithmic implementation, we refer the reader to the official documentation [9,13].

The necessary assumptions to identify causal effects in the selection-on-observables setting are the conditional independence assumption (CIA), exogeneity of the confounders, common support, and stable unit treatment value assumption (SUTVA). The CIA stipulates that treatment selection conditional on the set of so-called confounders is as good as random. By the exogeneity assumption, confounders need to be invariant to treatment assignment. The common support assumption demands that the probability of receiving a particular treatment is strictly bounded away from zero. Finally, SUTVA dictates that the observed outcome equals potential outcomes for the observed treatment state, ruling out interference between observational units or multiple versions of a treatment.

The causal parameters of interest comprise the individualized treatment effect (IATE), the group average treatment effect (GATE), and the average treatment effect (ATE). The IATE captures the expected causal impact of some treatment over another for a subpopulation, which is defined by a particular realization of confounders and further variables that are relevant for the heterogeneity analysis. To clarify, the number of comparisons that we take interest in in the multi-treatment setting with *k* treatments, which includes the control state, is k(k−1)/2. The GATE aggregates the IATEs to more coarse subpopulations, and the variables in the conditioning set are referred to as policy features. The conditioning feature(s) are (is) a low-dimensional subset of the set of confounders. Finally, the ATE is the expected causal impact for the entire population and hence obtained as a weighted average of the IATEs. For all parameters defined above, the conditioning set can be extended to include treatment group memberships. The causal parameters are then referred to as average treatment effect of the treated (ATET) and group average treatment effect of the treated (GATET), respectively.

The mcf is an instantiation of a causal forest, where splits in the tree growing process minimize the estimation error of the IATEs greedily. Ref. [9] showed that the expected mean-squared error (MSE) of the IATE can be decomposed into three parts: the two MSEs of estimating the conditional mean responses of the two treatments, which are causally compared, and the covariance of these two estimation errors (MCE). The estimates of the MSEs and the MCE are obtained as sample analogues. If no exact matches are found in all treatment leaves, the mcf uses the closest neighbor instead to compute the MCE. To guard against selection issues in finite samples, the mcf splitting rule seeks to assign individuals with different propensities of receiving a treatment to different partitions in the tree and hence prefers splits with high propensity score homogeneity. Estimates are then obtained as mean differences in the appropriate leaves. The mcf also builds upon the honesty principle, e.g., [8].

In the multiple treatment setting, one can grow the forests separately for each of the treatment comparisons or jointly for all unique treatment comparisons. For the latter case, the splits are chosen to minimize the sum of the estimated mean squared errors of the IATEs and the penalized propensity score heterogeneity. For inference, the mcf exploits that every causal forest can be written as a weighted sum of outcomes. Maintaining that observations are independent and identically distributed, ref. [9] derived an expression for the variance, which admits a utilization of standard non-parametric machine learners. The default method is k-Nearest Neighbor (k-NN) regression, but Nadaraya–Watson kernel estimation is also supported.

The *modified_causal_forest()* function in the mcf Python package implements the mcf. The user specifies treatment, outcome, confounders, policy variables, and the relevant resolutions of causal effect heterogeneity. Optionally, the user may override the defaults in the implementation—such as the grids for the parameter tuning in the forest growing process and the mode of parallelization. A detailed exposition of the functional inputs is given in the official documentation [13]. Whenever relevant, the documentation flags input arguments as critical for runtime management.

## 3. Empirical Studies

In this section, we demonstrate the functionality of the mcf. For three distinct research settings, we inquire to which extent the mcf matches previous estimation results on average treatment effects and provides novel insights on underlying effect heterogeneity.

### 3.1. Maternal Smoking during Pregnancy

Infants born at low birth weight (LBW) are more likely to experience health and development issues. Studies have found lower educational attainment, a poorer self-reported health status, and reduced employment and earnings for LBW infants, e.g., [14]. Study [15] is a well-known study that examines the impact of maternal smoking during pregnancy on birth weight, amongst other health outcomes. Adjusting for potential confounding factors, the authors of [15] estimated a negative impact of maternal smoking on birth weight. Later, ref. [16] deployed the [15] database to study multi-valued treatment effects. Ref. [16] found evidence for both (i) treatment heterogeneities and (ii) non-linearities in the effect sizes. We aimed to estimate the dose responses and to analyze IATEs along with GATEs to inform about effect heterogeneities.

We used the linked birth–infant death data in [15], which was made available to us by the author of [16]. The database compiles information for 511,940 births in Pennsylvania for the years 1989 to 1991—including details on birth weight, pregnancy, and parental characteristics. Smoking doses are defined as in [16]. We mapped the number of daily smoked cigarettes to a multivalued treatment variable, *T*, which takes on 6 distinct values: T∈{0,1,2,3,4,5} for the cigarette-bin-categories {0,1−5,6−10,11−15,16−20,21+}. The bins were chosen to capture the mass points in the distribution, which occur roughly every five cigarettes (a quarter of a US cigarette pack).

For identification, we stipulated the prototypical selection-on-observables setting. We note that this is not an innocuous assumption as, for example, [17] convincingly discussed. We informed our choice of confounders by [15,16]. We included parental socio-demographics (age, education, and race), pregnancy-related information (number of prenatal visits, adequacy of care, indicator if alcohol was consumed during the pregnancy, number of months elapsed since last pregnancy), birth-related information (month of birth, county of birth), and mother-related information (number of previous pregnancies, number of children born dead, indicator if born abroad). A detailed summary is given in Table S14 in the Supplementary Materials file.

We explored treatment response heterogeneities for different values of (i) maternal age, (ii) race, and (iii) number of care visits. The motivation for maternal age stems from the consideration that oocytes (eggs) and embryos from older mothers tend to be more susceptible to harmful environmental conditions such as smoking, e.g., [18]. Previous empirical studies have informed the other grouping features, including [19] and [20], respectively.

We took a random draw from the largest treatment group in the training data to speed up computations. The decrease in memory requirements and increase in computational speed was achieved at relatively low cost in terms of statistical precision.

**Figure 1:**
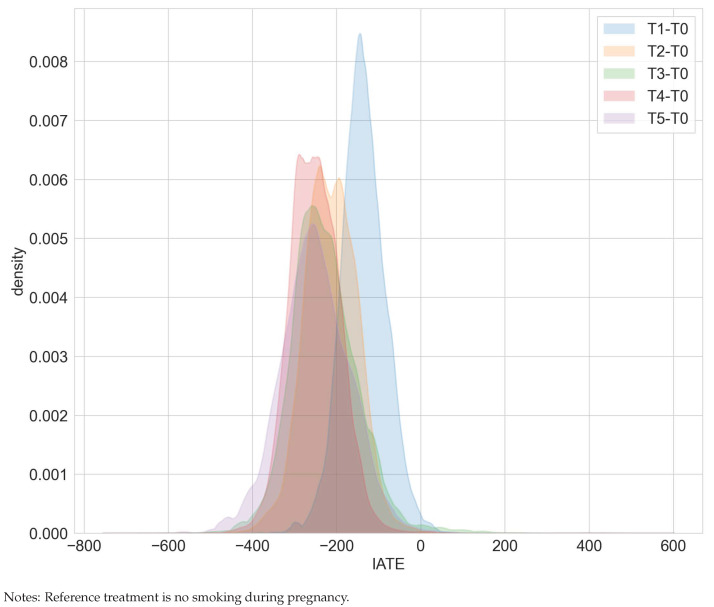
Distribution of IATEs in the maternal smoking during pregnancy study.

Overall, our estimation results are consistent with [15,16]. We found that smoking tends to reduce birth weight and that dose matters. Smoking more cigarettes is more detrimental in terms of birth weights (compare Table 1). The ATE for smoking one to five cigarettes over no cigarette consumption decreases from −136 to −252 for smoking 16 to 20 cigarettes over no cigarette consumption. The more detrimental effect of higher cigarette dosages is also suggested by the shifted distribution of the IATEs in Figure 1. However, none of the IATEs is significantly different from the corresponding ATEs.

**Figure 2:**
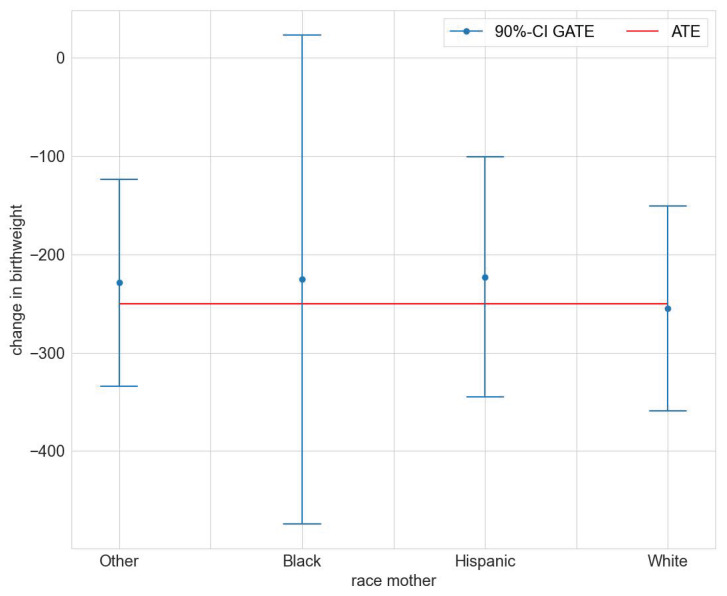
GATEs for maternal race in the maternal smoking during pregnancy study.

We found statistically significant GATEs for race, age, and number of prenatal visits (compare Tables S1, S3, and S5 in the Supplementary Materials file). Figure 2 illustrates the estimated GATEs for the different races. The effect is significantly different from zero for races Other, Hispanic, and White, but not so for Black. The estimated GATEs for race, age class, and number of prenatal visits are all not statistically significantly different from the ATE (compare Tables S2, S4, and S6 in the Supplementary Materials file). We conclude that the mcf does not indicate statistically significant effect heterogeneity.

### 3.2. Right Heart Catheterization

Right Heart Catheterization (RHC) is a surgical intervention widely used to monitor critically ill patients. In a seminal contribution, ref. [21] investigated the efficacy of this treatment measured by different outcomes (subsequent survival, length of stay, intensity of care, cost of care). Deploying propensity score matching, [21] found that RHC is positively associated with mortality, costs, and length of stay. The authors of [22,23,24] used alternative estimators and confirmed the findings in [21]. We matched previous results on the average effects of RHC on survival. Extending previous work, we added insights on effect heterogeneity, which the average treatment effect potentially masks.

The data we used are the same as in [21,22,23,24] and come from the SUPPORT prospective cohort study [25]. The data were made available by [24] (among others) and comprise information on 5735 critically ill and hospitalized adult patients between 1989 and 1994 in five medical centers spread throughout the US. Out of the 5735 patients, 2184 individuals received an RHC. In our analysis, we focused on survival within six months after treatment. As before, identification was achieved by stipulating unconfoundedness. In total, we included 55 features. For details refer to Table S15 in the Supplementary Materials file.

In the analysis of effect heterogeneity, we informed our choice of policy features by expert opinions who classified eight features as high-priority factors [22]. The high-priority factors include the nine primary disease categories, the estimated probability of surviving two months, the acute physiology and chronic health evaluation score, the Glasgow coma score indicator, age, an index of activities of daily living two weeks prior to admission, mean blood pressure, and an indicator for resuscitate status on the first day.

**Figure 3:**
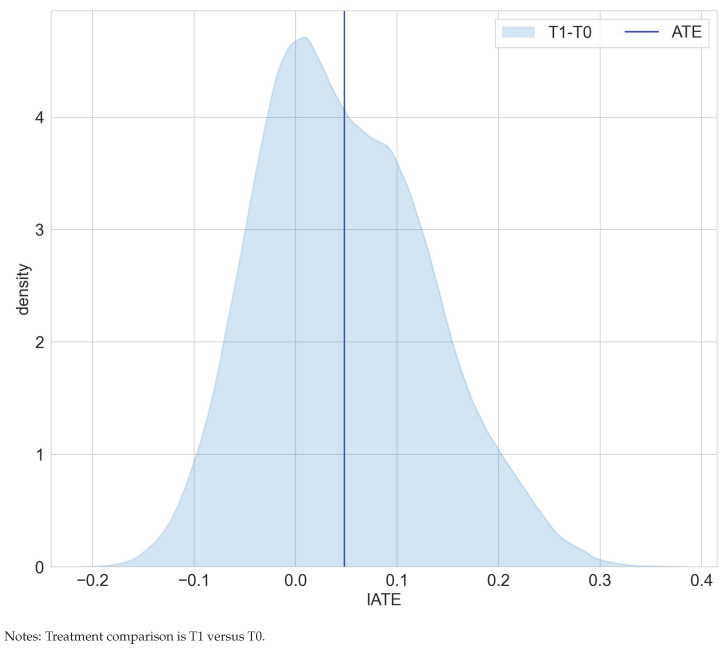
Distribution of IATEs in the RHC study.

**Figure 4:**
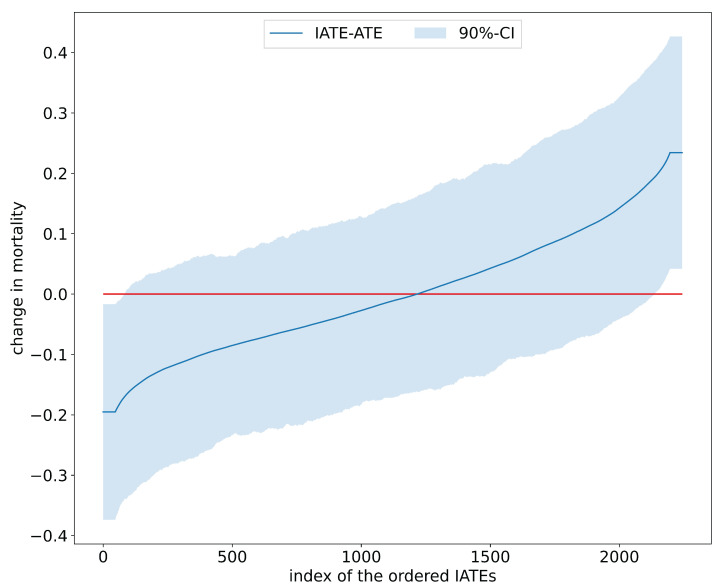
Sorted IATEs versus ATE in the RHC study.

Table 2 juxtaposes results on the estimated average effects of RHC on mortality after six months from [22] and the mcf. Findings for the ATE and ATET are congruent in terms of effect size and statistical significance and confirm that, on average, the RHC intervention decreases survival chances. Interestingly, as displayed in Figure 3, the distribution of the IATEs shows that there is a non-negligible mass left to zero. Abstracting from estimation uncertainty, some parts of the populations are estimated to benefit from the RHC intervention. An analysis of the difference of IATEs against the ATE confirms that subpopulations, which have IATEs at the tails of the distribution in Figure 4, have treatment effects that are statistically different from the ATE.

The mcf uncovered group effect heterogeneity, as Table 3 shows. Six out of eight policy features exhibit significant differences of the GATEs from the ATEs, pointing to effect heterogeneity in these policy features. Four policy features exhibit statistically significant GATEs.

**Figure 5:**
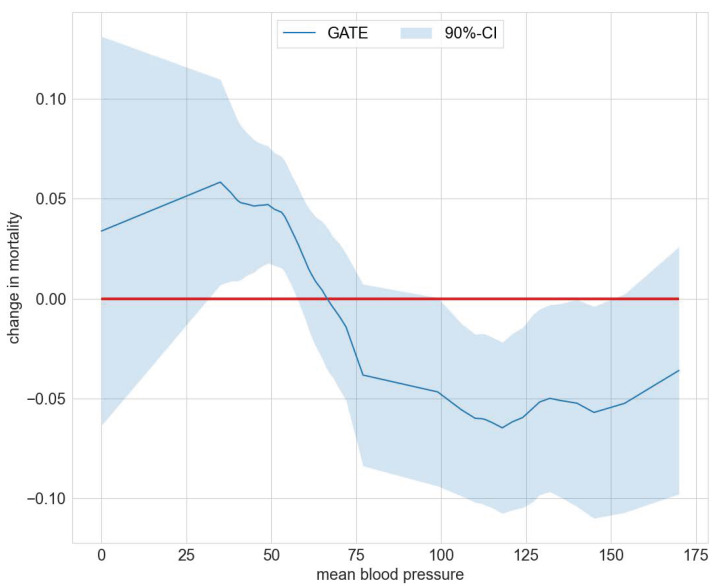
GATEs —ATE for mean blood pressure in the RHC study.

Exemplarily, Figure 5 summarizes the deviation of GATEs from the ATE for the policy feature blood pressure. The corresponding data for Figure 5 are included in Table S7 in the Supplementary Materials file. Figure 5 shows a significantly higher death risk for patients with extremely low diastolic blood pressure from 35 to 57 and lower death risk for a blood pressure from 106 to 145. Note that a diastolic blood pressure of zero may occur in cases of severe hypotension, stiff arteries in the elderly, diabetes, arteriovenous malformation, aortic dissection, or due to monitoring malfunction [26]. In the Supplementary Materials file we provide further results on effect heterogeneity in Tables S8 and S9. Patients with APACHE III scores ranging from 21 to 45 experience, on average, a significant increase in survival. Those with scores ranging from 55 to 66 have a significantly lower survival probability. For the policy feature summarizing the patient’s primary disease, Table S9 displays a significantly higher death risk than the average for patients with non-traumatic coma.

### 3.3. The Workforce Investment Act Programs

The Workforce Investment Act of 1998 (WIA) is the central federal workforce development legislation in the United States, which succeeded the Job Training Partnership Act (JTPA) and became operational from 1999 to 2000. The WIA programs provide services for education and training to increase the labor market prospects of adults, displaced workers, and youth. Participation in WIA services often starts in so-called one-stop centers, which are spread out over the US. In total, there are 3000 one-stop centers. More details on the WIA are summarized in [27]. Individuals participate in WIA-funded services voluntarily. The services for adults and dislocated workers fall into four categories: self-service core services, staff-assisted core services, intensive services, and training services. There are no eligibility criteria for the core services [28]. Individuals usually set up an individual training account to participate in a training service and select training and provider. Caseworkers may encourage or discourage participation in specific programs. Unlike in some European countries, caseworkers cannot sanction the clients [28,29]. The WIA was replaced by the Workforce Innovation and Opportunity Act (WIOA) in 2013. Neither the basic set of services nor eligibility were much affected by the new legislation [28,30].

Previous studies found a positive impact of receivers of training over the core and/or intensive services for WIA participants [28], and for WIA participants over Employment Service (ES) participants [31] or unemployment insurance claimants and ES participants. The authors of [30] found relevant heterogeneity in levels of program participation for the examined WIA population. For identification, refs. [28,31] relied upon a selection-on-observables framework and [30] on the invariance of conditional distributions. The authors of [28] added an analysis where selection is on unobservables but maintained bias stability across time and found similar results.

We used the database from [30]. The database synthesizes information on 85,440 individuals served by WIA and WIOA programs in California between 2012 and 2016. Treatment takes four values, T∈{1,2,3,4}, where 1 indicates core services, 2 intensive service, 3 basic/general training, and 4 occupational training service. Following [30], we defined the outcome as the differences in average earnings four quarters after exiting the program and three quarters before entering it. As before, identification was achieved by stipulating conditional independence of treatment assignment and potential outcomes controlling for all observables. In total, we included 24 features. For details refer to Table S16.

Table 4 juxtaposes results on the estimated average effects from [30]—columns two to four—and the mcf—columns five to seven. Point estimates for the two estimators are aligned. The effects range from $317 to $1957 for the mcf and from $99 to $1739 for the doubly robust GMM estimation method based on inverse probability weighting applied in [30]. We observed the largest effect for the treatment pair occupational training service (T4) and core services (T1). Participating in occupational training compared to core services increased earnings on average by $1957 ([30] estimated $1739). Note that the estimated weights-based standard errors of the mcf are larger than the bootstrapped standard errors of [30], which were based on resampling estimates of the influence function.

**Figure 6:**
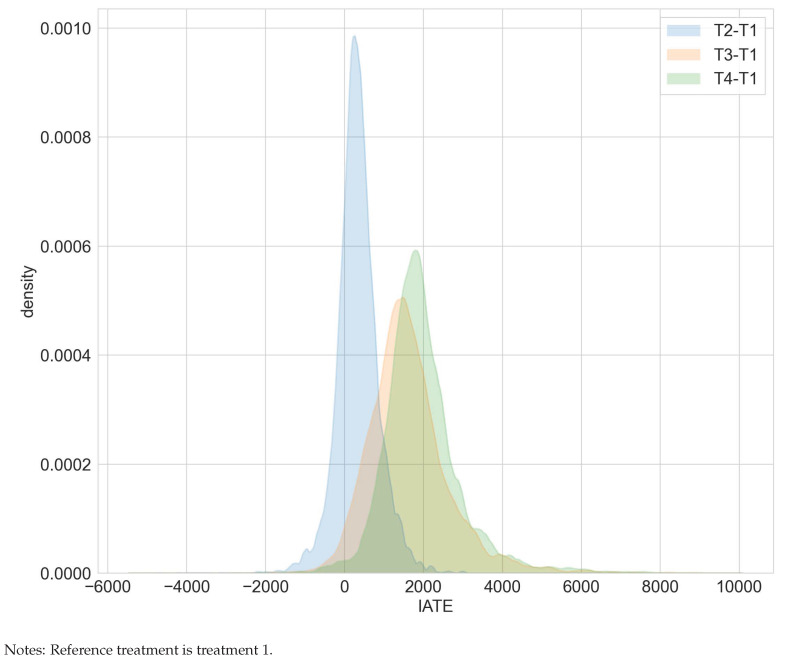
Distribution of IATEs in the WIA programs study.

The superiority of occupational training over core services is also reflected in Figure 6. Ignoring estimation uncertainty, the estimated IATEs for comparing occupational training (T4) versus core services (T1) are prevailingly positive.

**Figure 7:**
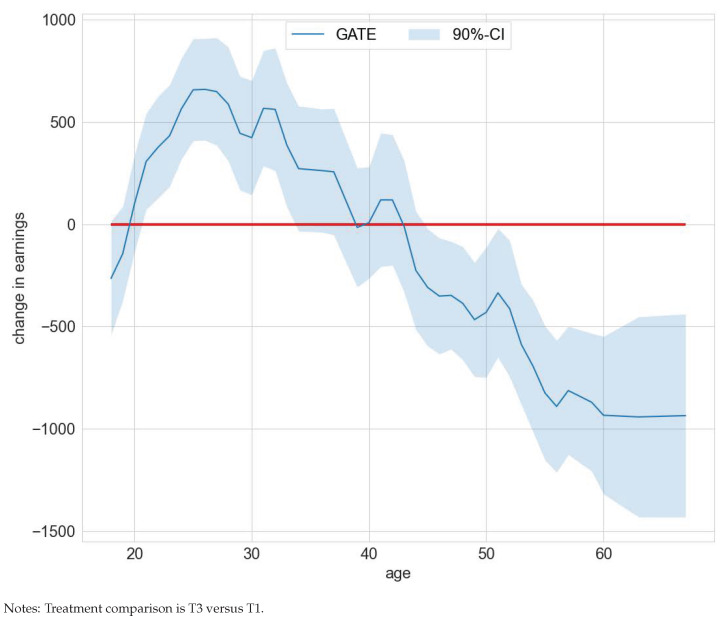
GATEs —ATE by age in the WIA programs study.

Our group heterogeneity analysis focused on two policy features—claim to unemployment compensation and age. The authors of [30] showed that unemployment compensation status is an important confounding feature and hence may give rise to effect heterogeneity. Indeed, the GATE deviates statistically significantly from the ATE for the policy feature unemployment compensation. The deviations are negative for subjects with a claim to unemployment for T3 versus T1, T3 versus T2, and positive for T4 versus T3. Contrariwise, the deviations are positive for subjects without a claim to unemployment for T3 versus T1, T3 versus T2, and negative for T4 versus T2. The results also hint at meaningful effect heterogeneity for the policy feature age as measured by a significant deviation of the GATE from the ATE. For example, when comparing treatment groups T3 versus T1, the GATE deviates positively from the ATE for ages 21 to 33 and negatively for ages 45 to 67 (compare Figure 7). This hints at an optimal assignment rule that should target clients of different ages and unemployment compensation statuses differently when resource or capacity constraints are binding. Detailed results on the GATEs and GATEs minus the ATEs for both policy features age and claim to unemployment compensation are included in Tables S10–S13 in the Supplementary Materials file.

## 4. Discussion

The modified causal forest (mcf) matched results on aggregate treatment effects estimation and provided novel insights on underlying effect heterogeneity. The distilled effect heterogeneity exhibited meaningful patterns for the RHC and WIA studies in that some populations benefited more or less than the average from the treatment intervention. The generated insights hint at more efficient targeting rules when resource or capacity constraints are binding. Mirroring the burgeoning literature in optimal policy learning, since version 0.1.0 the mcf includes a functionality to learn minimax regret optimal treatment assignments when the policy class is restricted to decision trees.

The mcf is under ongoing development to incorporate new functionalities. Since version 0.2.0, the mcf accommodates continuous treatment effects estimation as an experimental feature. In addition, the mcf provides statistics on balancing and common support to evaluate the quality of the obtained causal parameters. There is ongoing research to formalize the underlying statistics and provide critical values for practitioners.

## Figures and Tables

**Table 1:** ATEs in the maternal smoking during pregnancy study.

TC	[16]	mcf
T1-T0	−146 *	−136 *
T2-T0	−217 *	−213 *
T3-T0	−254 *	−228 *
T4-T0	−255 *	−252 *
T5-T0	−252 *	−250 *
T2-T1	−71 *	−77 *
T3-T1	−108 *	−92 *
T4-T1	−109 *	−115 *
T5-T1	−106 *	−114
T3-T2	−37	−15
T4-T2	−38 *	−38
T5-T2	−35 *	−37
T4-T3	−1	−23
T5-T3	2	−22
T5-T4	3	1

Notes: TC denotes treatment comparison; estimates from [16] are printed in column two, estimates from the mcf in column three. * denotes significance at the 5% level.

**Table 2:** ATEs and ATETs in the RHC study.

Method	Estimand	Point Estimate	*p*-Value
ps match	ATET	0.063	0.005
gm match	ATET	0.046	0.037
mcf	ATE	0.048	0.013
mcf	ATET	0.065	0.001

Notes: ps match and gm match refer to propensity score and genetic matching applied in [22], respectively. ATE stands for the average treatment effect, ATET denotes the average treatment effect on the treated.

**Table 3:** GATE results for the RHC study.

Feature	Evaluation Points	Number of Significant GATEs	Number of Significant GATEs-ATEs
adld3pc	27	0	0
age	50	3	9
aps1	49	15	26
cat1	9	0	1
dnr1	2	0	0
meanbp1	49	15	32
scoma1	11	0	2
surv2md1	50	1	1

Notes: The significance level was set to 10%; adld3pc is the index of activities of daily living two weeks prior to admission; aps1 is the acute physiology and chronic health evaluation score; cat1 are the nine primary disease classes; dnr1 is an indicator for resuscitate status on the first day; meanbp1 is the mean blood pressure; scoma1 is the Glasgow coma score; surv2md1 is the probability of surviving two months based on support model estimation.

**Table 4:** ATEs in the WIA programs study.

TC	ATE	[30] SE	*p*-Value	ATE	mcf SE	*p*-Value
T2-T1	99	41	0.02	335	63	0.00
T3-T1	1273	56	0.00	1640	87	0.00
T4-T1	1739	85	0.00	1957	126	0.00
T3-T2	1174	53	0.00	1305	81	0.00
T4-T2	1640	82	0.00	1622	122	0.00
T4-T3	466	89	0.00	317	136	0.02

Notes: TC stands for treatment comparison, ATE for average treatment effects, SE for standard errors.

## Data Availability

All data are made available on GitHub [12].

## Supplementary Materials

The supporting information can be downloaded at https://www.mdpi.com/article/10.3390/e24081039/s1. Tables S1–S13 compile results from the group average treatment affects (GATEs) analysis. Tables S14–S16 provide an exhaustive variable description of the three data sets used in the empirical analysis.
