# Supplementary material for: High Resolution Treatment Effects Estimation: Uncovering Effect Heterogeneities with the Modified Causal Forest
Source: Entropy (Basel). 2022 Jul 28;24(8):1039. doi: 10.3390/e24081039 (PMC9407165; doi:10.3390/e24081039)
Supplement: Supplementary file 1 [file entropy-24-01039-s001.zip › entropy-1796223-supplementary.pdf]

Supplementary Material for "High Resolution Treatment Effects Estimation: Uncovering Effect Heterogeneities with the Modified Causal Forest"

Hugo Bodory*, Hannah Busshoff†, Michael Lechner‡

July 22, 2022

*Vice-President's Board (Research & Faculty), University of St. Gallen, Dufourstrasse 50, 9000 St. Gallen, hugo.bodory@unisg.ch
†Swiss Institute for Empirical Research, University of St. Gallen, Varnbüelstrasse 14, 9000 St. Gallen, hannah.busshoff@unisg.ch
‡Swiss Institute for Empirical Research, University of St. Gallen, Varnbüelstrasse 14, 9000 St. Gallen, michael.lechner@unisg.ch

Table S1: GATEs for maternal age in the maternal smoking during pregnancy study

	**1 vs. 0**	**2 vs. 0**	**3 vs. 0**	**4 vs. 0**	**5 vs. 0**	**2 vs. 1**	**3 vs. 1**	**4 vs. 1**
14	-83.05	-123.68	-121.37	-160.55	-174.73	-40.63	-38.32	-77.5
	[-248.18, 82.07]	[-267.66, 20.29]	[-443.51, 200.76]	[-328.12, 7.01]	[-492.02, 142.56]	[-183.14, 101.88]	[-359.8, 283.16]	[-243.8, 88.81]
15	-98.09	-152.1	-159.89	-190.59	-191.15	-54.01	-61.8	-92.5
	[-191.57, -4.61]	[-234.91, -69.29]	[-323.59, 3.8]	[-287.62, -93.56]	[-399.09, 16.78]	[-132.49, 24.47]	[-223.35, 99.75]	[-185.87, 0.86]
20	-123.22	-195.1	-213.09	-236.69	-244.16	-71.88	-89.87	-113.47
	[-178.57, -67.88]	[-240.78, -149.42]	[-296.93, -129.25]	[-290.78, -182.6]	[-365.25, -123.08]	[-122.55, -21.21]	[-176.53, -3.21]	[-171.83, -55.11]
25	-139.61	-224.12	-238.61	-260.11	-260.53	-84.51	-98.99	-120.49
	[-203.43, -75.8]	[-272.06, -176.18]	[-333.42, -143.8]	[-318.59, -201.62]	[-393.55, -127.51]	[-155.79, -13.23]	[-207.49, 9.5]	[-199.25, -41.73]
30	-152.83	-235.87	-250.33	-273.53	-264.97	-83.04	-97.5	-120.71
	[-248.15, -57.5]	[-307.25, -164.49]	[-394.05, -106.61]	[-355.94, -191.13]	[-429.05, -100.88]	[-191.71, 25.62]	[-262.94, 67.93]	[-236.91, -4.5]
35	-162.66	-244.81	-263.64	-281.81	-266.97	-82.15	-100.98	-119.14
	[-281.93, -43.4]	[-335.45, -154.18]	[-448.04, -79.24]	[-385.32, -178.3]	[-467.72, -66.23]	[-218.72, 54.42]	[-311.79, 109.83]	[-264.58, 26.29]
40	-167.54	-244.52	-258.16	-279.77	-257.89	-76.98	-90.63	-112.23
	[-285.53, -49.55]	[-334.26, -154.78]	[-441.06, -75.27]	[-381.43, -178.11]	[-451.91, -63.87]	[-209.17, 55.21]	[-297.68, 116.43]	[-252.78, 28.32]
45	-166.25	-243.45	-243.45	-276.53	-238.33	-77.2	-77.19	-110.28
	[-319.79, -12.72]	[-356.1, -130.8]	[-445.63, -41.26]	[-396.42, -156.63]	[-439.27, -37.38]	[-229.85, 75.45]	[-304.11, 149.72]	[-268.35, 47.79]

Table S1 continued from previous page

	**5 vs. 1**	**3 vs. 2**	**4 vs. 2**	**5 vs. 2**	**4 vs. 3**	**5 vs. 3**	**5 vs. 4**
14	-91.68	2.31	-36.87	-51.05	-39.18	-53.36	-14.18
	[-408.31, 224.95]	[-308.84, 313.46]	[-182.19, 108.46]	[-357.18, 255.08]	[-361.92, 283.56]	[-473.79, 367.07]	[-332.09, 303.73]
15	-93.06	-7.79	-38.49	-39.05	-30.7	-31.26	-0.56
	[-299.31, 113.18]	[-163.4, 147.82]	[-121.16, 44.18]	[-240.69, 162.58]	[-194.33, 132.92]	[-277.42, 214.9]	[-208.44, 207.32]
20	-120.94	-17.99	-41.59	-49.07	-23.6	-31.07	-7.47
	[-244.0, 2.12]	[-98.82, 62.84]	[-90.88, 7.7]	[-168.09, 69.96]	[-109.46, 62.26]	[-169.31, 107.16]	[-129.97, 115.02]
25	-120.92	-14.49	-35.98	-36.41	-21.5	-21.93	-0.43
	[-264.02, 22.17]	[-114.47, 85.5]	[-102.53, 30.57]	[-173.17, 100.34]	[-126.95, 83.95]	[-181.28, 137.43]	[-141.23, 140.37]
30	-112.14	-14.46	-37.66	-29.1	-23.2	-14.64	8.57
	[-295.55, 71.27]	[-167.36, 138.44]	[-135.19, 59.87]	[-201.28, 143.08]	[-181.55, 135.14]	[-227.25, 197.98]	[-168.47, 185.6]
35	-104.31	-18.83	-36.99	-22.16	-18.16	-3.33	14.83
	[-329.56, 120.94]	[-214.87, 177.21]	[-160.05, 86.06]	[-233.65, 189.33]	[-220.48, 184.15]	[-268.88, 262.22]	[-202.49, 232.15]
40	-90.35	-13.64	-35.25	-13.37	-21.61	0.27	21.88
	[-307.29, 126.59]	[-206.01, 178.72]	[-153.1, 82.6]	[-216.34, 189.6]	[-219.81, 176.6]	[-257.78, 258.33]	[-186.63, 230.39]
45	-72.08	0.0	-33.08	5.12	-33.08	5.12	38.2
	[-297.89, 153.73]	[-201.51, 201.51]	[-151.83, 85.68]	[-195.15, 205.39]	[-238.73, 172.57]	[-256.22, 266.45]	[-166.23, 242.63]

Note: The table summarizes the Group Average Treatment Effects (GATEs) for the policy feature age in the birthweight study. The evaluation points are given in the leftmost column. 90 % confidence intervals are given below the point estimates in square brackets. Value labels are given in Table 14.

Table S2: GATEs - ATE for maternal age in the maternal smoking during pregnancy study

	**1 vs. 0**	**2 vs. 0**	**3 vs. 0**	**4 vs. 0**	**5 vs. 0**	**2 vs. 1**	**3 vs. 1**	**4 vs. 1**
14	53.08	89.8	107.05	91.06	75.56	36.73	53.98	37.98
	[-125.91, 232.06]	[-61.66, 241.26]	[-220.85, 434.96]	[-83.71, 265.83]	[-249.45, 400.57]	[-126.07, 199.52]	[-279.32, 387.27]	[-146.7, 222.66]
15	38.04	61.38	68.53	61.02	59.14	23.35	30.5	22.98
	[-69.72, 145.8]	[-27.78, 150.55]	[-104.99, 242.06]	[-41.74, 163.78]	[-152.06, 270.34]	[-79.84, 126.53]	[-150.64, 211.63]	[-92.16, 138.12]
20	12.91	18.39	15.34	14.92	6.13	5.48	2.43	2.01
	[-49.95, 75.77]	[-30.66, 67.44]	[-77.24, 107.91]	[-40.94, 70.78]	[-105.1, 117.36]	[-59.05, 70.01]	[-99.2, 104.06]	[-67.83, 71.86]
25	-3.48	-10.64	-10.18	-8.5	-10.24	-7.15	-6.7	-5.01
	[-50.62, 43.65]	[-47.31, 26.04]	[-79.68, 59.32]	[-50.62, 33.62]	[-94.91, 74.43]	[-55.79, 41.49]	[-83.19, 69.79]	[-57.88, 47.85]
30	-16.7	-22.38	-21.9	-21.92	-14.67	-5.69	-5.21	-5.23
	[-87.98, 54.59]	[-77.51, 32.74]	[-128.23, 84.42]	[-84.34, 40.5]	[-134.75, 105.4]	[-81.23, 69.86]	[-123.41, 113.0]	[-86.25, 75.8]
35	-26.53	-31.33	-35.22	-30.2	-16.68	-4.79	-8.68	-3.66
	[-123.59, 70.52]	[-106.6, 43.94]	[-185.18, 114.75]	[-115.07, 54.68]	[-174.17, 140.81]	[-110.7, 101.11]	[-176.14, 158.77]	[-116.6, 109.28]
40	-31.41	-31.03	-29.74	-28.16	-7.6	0.38	1.67	3.25
	[-130.33, 67.51]	[-107.43, 45.36]	[-180.17, 120.69]	[-113.3, 56.98]	[-165.67, 150.48]	[-105.21, 105.96]	[-165.49, 168.83]	[-108.83, 115.33]
45	-30.12	-29.96	-15.02	-24.92	11.97	0.16	15.1	5.2
	[-176.04, 115.8]	[-138.17, 78.24]	[-203.31, 173.28]	[-140.05, 90.21]	[-181.48, 205.41]	[-142.34, 142.65]	[-194.78, 224.99]	[-142.62, 153.03]

Table S2 continued from previous page

	**5 vs. 1**	**3 vs. 2**	**4 vs. 2**	**5 vs. 2**	**4 vs. 3**	**5 vs. 3**	**5 vs. 4**
14	22.49	17.25	1.26	-14.24	-16.0	-31.49	-15.5
	[-307.96, 352.93]	[-302.11, 336.61]	[-156.89, 159.4]	[-330.63, 302.15]	[-347.04, 315.05]	[-461.24, 398.26]	[-343.68, 312.69]
15	21.1	7.15	-0.37	-2.24	-7.52	-9.39	-1.88
	[-196.39, 238.59]	[-163.57, 177.87]	[-98.32, 97.58]	[-211.15, 206.66]	[-185.72, 170.68]	[-265.91, 247.13]	[-216.94, 213.18]
20	-6.78	-3.05	-3.47	-12.26	-0.42	-9.21	-8.79
	[-125.64, 112.09]	[-96.77, 90.66]	[-61.2, 54.26]	[-124.43, 99.92]	[-97.87, 97.03]	[-146.13, 127.72]	[-124.11, 106.53]
25	-6.76	0.46	2.14	0.4	1.68	-0.06	-1.74
	[-97.25, 83.74]	[-70.07, 70.98]	[-41.66, 45.94]	[-85.12, 85.91]	[-71.82, 75.19]	[-103.97, 103.85]	[-89.73, 86.25]
30	2.02	0.48	0.46	7.71	-0.02	7.23	7.25
	[-128.69, 132.74]	[-108.75, 109.7]	[-66.78, 67.71]	[-114.94, 130.36]	[-113.1, 113.06]	[-145.44, 159.91]	[-118.85, 133.35]
35	9.85	-3.89	1.13	14.65	5.02	18.54	13.52
	[-164.37, 184.08]	[-159.73, 151.96]	[-93.74, 96.0]	[-148.45, 177.74]	[-155.68, 165.72]	[-189.85, 226.92]	[-154.23, 181.26]
40	23.81	1.3	2.87	23.44	1.58	22.14	20.56
	[-150.26, 197.88]	[-153.6, 156.19]	[-89.93, 95.68]	[-138.89, 185.77]	[-157.81, 160.97]	[-185.57, 229.85]	[-146.06, 187.19]
45	42.09	14.94	5.05	41.93	-9.9	26.98	36.88
	[-172.43, 256.6]	[-170.71, 200.6]	[-105.72, 115.81]	[-148.95, 232.81]	[-199.68, 179.88]	[-218.33, 272.3]	[-158.0, 231.77]

Note: The table summarizes the difference of Group Average Treatment Effects (GATEs) minus the Average Treatment Effects (ATEs) for the policy feature age in the birthweight study. The different values of the policy features are given in the leftmost column. 90 % confidence intervals are given below the point estimates in square brackets. Value labels are given in Table 14.

Table S3: GATEs for maternal race in the maternal smoking during pregnancy study

	**1 vs. 0**	**2 vs. 0**	**3 vs. 0**	**4 vs. 0**	**5 vs. 0**	**2 vs. 1**	**3 vs. 1**	**4 vs. 1**
0	-137.88	-212.13	-215.79	-243.58	-228.83	-74.25	-77.92	-105.7
	[-195.31, -80.44]	[-258.09, -166.16]	[-312.01, -119.58]	[-297.62, -189.53]	[-334.35, -123.32]	[-131.72, -16.78]	[-180.12, 24.29]	[-169.81, -41.59]
1	-138.17	-184.97	-221.58	-205.81	-225.29	-46.79	-83.4	-67.64
	[-242.49, -33.86]	[-279.67, -90.27]	[-463.65, 20.5]	[-327.64, -83.98]	[-473.94, 23.37]	[-135.44, 41.85]	[-323.17, 156.37]	[-184.82, 49.55]
2	-127.98	-191.9	-205.9	-225.76	-222.9	-63.92	-77.92	-97.78
	[-187.5, -68.46]	[-243.98, -139.82]	[-326.44, -85.36]	[-288.65, -162.88]	[-344.95, -100.85]	[-114.82, -13.02]	[-197.95, 42.11]	[-159.69, -35.87]
3	-135.78	-218.49	-229.85	-259.7	-254.98	-82.71	-94.07	-123.92
	[-186.91, -84.65]	[-256.4, -180.59]	[-303.93, -155.78]	[-305.39, -214.0]	[-359.25, -150.71]	[-142.38, -23.05]	[-181.31, -6.83]	[-188.81, -59.02]

Table S3 continued from previous page

	5 vs. 1	3 vs. 2	4 vs. 2	5 vs. 2	4 vs. 3	5 vs. 3	5 vs. 4
0	-90.95	-3.67	-31.45	-16.71	-27.78	-13.04	14.75
	[-201.96, 20.05]	[-99.9, 92.56]	[-85.53, 22.62]	[-122.24, 88.83]	[-128.12, 72.56]	[-148.24, 122.17]	[-94.55, 124.04]
1	-87.11	-36.61	-20.84	-40.32	15.76	-3.71	-19.48
	[-333.53, 159.3]	[-272.35, 199.14]	[-129.56, 87.87]	[-282.82, 202.18]	[-232.13, 263.66]	[-333.01, 325.59]	[-273.8, 234.85]
2	-94.92	-14.0	-33.86	-31.0	-19.86	-17.0	2.86
	[-216.47, 26.64]	[-130.52, 102.53]	[-88.66, 20.94]	[-149.09, 87.09]	[-141.6, 101.87]	[-177.51, 143.51]	[-120.37, 126.1]
3	-119.2	-11.36	-41.2	-36.49	-29.84	-25.13	4.71
	[-233.2, -5.2]	[-91.57, 68.85]	[-96.29, 13.88]	[-145.2, 72.22]	[-114.01, 54.32]	[-151.1, 100.84]	[-106.95, 116.38]

Note: The table summarizes the Group Average Treatment Effects (GATEs) for the policy feature race in the birthweight study. The evaluation points are given in the leftmost column. 90 % confidence intervals are given below the point estimates in square brackets. Value labels are given in Table 14.

Table S4: GATEs - ATE for maternal race in the maternal smoking during pregnancy study

	**1 vs. 0**	**2 vs. 0**	**3 vs. 0**	**4 vs. 0**	**5 vs. 0**	**2 vs. 1**	**3 vs. 1**	**4 vs. 1**
0	-1.75	1.36	12.63	8.03	21.46	3.11	14.38	9.78
	[-53.02, 49.53]	[-39.66, 42.38]	[-65.93, 91.2]	[-38.03, 54.09]	[-63.16, 106.09]	[-45.87, 52.09]	[-68.62, 97.38]	[-43.49, 63.05]
1	-2.04	28.52	6.85	45.8	25.01	30.56	8.9	47.84
	[-119.71, 115.62]	[-71.34, 128.38]	[-226.91, 240.61]	[-77.1, 168.7]	[-220.79, 270.81]	[-79.8, 140.93]	[-229.54, 247.33]	[-83.74, 179.42]
2	8.15	21.58	22.53	25.85	27.4	13.44	14.38	17.7
	[-57.12, 73.42]	[-31.56, 74.73]	[-87.09, 132.15]	[-35.55, 87.24]	[-87.4, 142.19]	[-48.7, 75.57]	[-99.87, 128.63]	[-51.62, 87.02]
3	0.35	-5.01	-1.43	-8.09	-4.69	-5.35	-1.77	-8.44
	[-20.32, 21.01]	[-22.53, 12.51]	[-42.36, 39.5]	[-29.65, 13.47]	[-47.8, 38.42]	[-24.79, 14.08]	[-43.56, 40.01]	[-31.58, 14.71]

Table S4 continued from previous page

	5 vs. 1	3 vs. 2	4 vs. 2	5 vs. 2	4 vs. 3	5 vs. 3	5 vs. 4
0	23.21	11.27	6.67	20.1	-4.6	8.83	13.43
	[-65.54, 111.96]	[-65.82, 88.36]	[-36.82, 50.16]	[-63.15, 103.36]	[-84.48, 75.28]	[-98.04, 115.7]	[-72.42, 99.28]
1	27.05	-21.67	17.28	-3.51	38.95	18.16	-20.79
	[-223.2, 277.3]	[-251.84, 208.5]	[-98.65, 133.21]	[-245.9, 238.88]	[-202.11, 280.01]	[-303.44, 339.75]	[-273.55, 231.96]
2	19.25	0.94	4.26	5.81	3.32	4.87	1.55
	[-99.97, 138.47]	[-106.84, 108.73]	[-53.79, 62.31]	[-107.23, 118.85]	[-108.76, 115.4]	[-143.36, 153.09]	[-115.6, 118.69]
3	-5.04	3.58	-3.08	0.32	-6.66	-3.26	3.4
	[-48.96, 38.88]	[-36.74, 43.9]	[-23.46, 17.3]	[-42.21, 42.85]	[-48.89, 35.57]	[-59.63, 53.1]	[-40.95, 47.75]

Note: The table summarizes the difference of Group Average Treatment Effects (GATEs) minus the Average Treatment Effects (ATEs) for the policy feature race in the birthweight study. 90 % confidence intervals are given below the point estimates in square brackets. The evaluation points are given in the leftmost column. Value labels are given in Table 14.

Table S5: GATEs for the number of prenatal visits in the maternal smoking during pregnancy study

	**1 vs. 0**	**2 vs. 0**	**3 vs. 0**	**4 vs. 0**	**5 vs. 0**	**2 vs. 1**	**3 vs. 1**	**4 vs. 1**
-99	-156.33	-211.72	-227.18	-257.57	-184.39	-55.39	-70.85	-101.24
	[-372.32, 59.66]	[-394.29, -29.14]	[-613.24, 158.88]	[-454.81, -60.33]	[-516.78, 148.01]	[-251.44, 140.67]	[-463.47, 321.77]	[-311.03, 108.55]
0	-111.0	-188.58	-226.22	-237.9	-213.6	-77.59	-115.23	-126.9
	[-296.09, 74.09]	[-345.61, -31.55]	[-554.73, 102.29]	[-406.65, -69.15]	[-476.73, 49.54]	[-227.91, 72.74]	[-440.59, 210.13]	[-289.43, 35.62]
5	-123.02	-209.53	-209.42	-250.21	-242.29	-86.51	-86.41	-127.19
	[-247.22, 1.19]	[-306.09, -112.96]	[-390.16, -28.68]	[-356.56, -143.85]	[-428.44, -56.13]	[-205.29, 32.27]	[-279.93, 107.12]	[-254.06, -0.32]
10	-142.2	-217.74	-235.58	-253.72	-258.97	-75.54	-93.38	-111.52
	[-213.54, -70.85]	[-272.21, -163.27]	[-345.01, -126.14]	[-319.43, -188.02]	[-405.05, -112.9]	[-156.58, 5.5]	[-218.18, 31.43]	[-200.51, -22.54]
16	-144.5	-212.94	-226.66	-248.77	-250.6	-68.44	-82.16	-104.27
	[-218.73, -70.27]	[-271.35, -154.52]	[-344.47, -108.86]	[-319.81, -177.73]	[-407.53, -93.68]	[-152.92, 16.05]	[-214.84, 50.52]	[-197.93, -10.6]

Table S5 continued from previous page

	5 vs. 1	3 vs. 2	4 vs. 2	5 vs. 2	4 vs. 3	5 vs. 3	5 vs. 4
-99	-28.06	-15.46	-45.85	27.33	-30.39	42.79	73.18
	[-368.05, 311.94]	[-390.74, 359.81]	[-221.04, 129.34]	[-292.47, 347.14]	[-413.02, 352.24]	[-424.1, 509.69]	[-255.22, 401.58]
0	-102.6	-37.64	-49.32	-25.01	-11.68	12.63	24.3
	[-361.79, 156.59]	[-347.9, 272.61]	[-178.98, 80.35]	[-264.97, 214.94]	[-328.02, 304.67]	[-362.66, 387.92]	[-223.48, 272.09]
5	-119.27	0.1	-40.68	-32.76	-40.78	-32.87	7.92
	[-317.86, 79.32]	[-176.95, 177.16]	[-140.65, 59.29]	[-215.34, 149.82]	[-223.36, 141.8]	[-270.94, 205.2]	[-180.02, 195.86]
10	-116.77	-17.84	-35.98	-41.23	-18.15	-23.39	-5.25
	[-274.69, 41.15]	[-133.82, 98.15]	[-112.1, 40.14]	[-192.28, 109.82]	[-139.82, 103.52]	[-201.79, 155.0]	[-160.7, 150.21]
16	-106.1	-13.73	-35.83	-37.66	-22.1	-23.94	-1.83
	[-274.48, 62.28]	[-138.25, 110.8]	[-117.53, 45.87]	[-199.7, 124.37]	[-153.03, 108.82]	[-215.56, 167.68]	[-168.83, 165.16]

Note: The table summarizes the Group Average Treatment Effects (GATEs) for the policy feature number of prenatal visits in the birthweight study. 90 % confidence intervals are given below the point estimates in square brackets. The evaluation points are given in the leftmost column. Value labels are given in Table 14.

Table S6: GATEs - ATE for the number of prenatal visits in the maternal smoking during pregnancy study

	**1 vs. 0**	**2 vs. 0**	**3 vs. 0**	**4 vs. 0**	**5 vs. 0**	**2 vs. 1**	**3 vs. 1**	**4 vs. 1**
-99	-20.2	1.77	1.25	-5.96	65.91	21.97	21.45	14.24
	[-241.43, 201.03]	[-183.57, 187.11]	[-383.26, 385.76]	[-207.46, 195.55]	[-283.43, 415.24]	[-183.72, 227.66]	[-373.27, 416.17]	[-206.12, 234.6]
0	25.13	24.9	2.2	13.71	36.7	-0.23	-22.93	-11.42
	[-169.12, 219.39]	[-136.96, 186.76]	[-331.44, 335.84]	[-162.18, 189.61]	[-253.24, 326.63]	[-168.89, 168.43]	[-359.92, 314.06]	[-193.59, 170.75]
5	13.11	3.96	19.0	1.4	8.01	-9.15	5.89	-11.71
	[-115.06, 141.29]	[-95.75, 103.67]	[-169.13, 207.14]	[-109.72, 112.53]	[-195.26, 211.28]	[-136.15, 117.84]	[-198.02, 209.8]	[-147.85, 124.43]
10	-6.07	-4.25	-7.15	-2.11	-8.68	1.82	-1.08	3.96
	[-54.7, 42.56]	[-42.38, 33.87]	[-80.4, 66.1]	[-44.69, 40.46]	[-86.94, 69.59]	[-46.31, 49.94]	[-80.0, 77.83]	[-47.76, 55.68]
16	-8.37	0.55	1.76	2.84	-0.31	8.92	10.14	11.21
	[-62.61, 45.87]	[-42.54, 43.64]	[-81.93, 85.45]	[-46.28, 51.96]	[-92.75, 92.13]	[-45.48, 63.32]	[-79.9, 100.17]	[-48.07, 70.5]

Table S6 continued from previous page

	5 vs. 1	3 vs. 2	4 vs. 2	5 vs. 2	4 vs. 3	5 vs. 3	5 vs. 4
-99	86.11	-0.52	-7.73	64.14	-7.21	64.66	71.87
	[-274.43, 446.65]	[-376.31, 375.26]	[-192.04, 176.58]	[-275.57, 403.85]	[-391.22, 376.81]	[-413.77, 543.09]	[-276.92, 420.65]
0	11.57	-22.7	-11.19	11.79	11.51	34.49	22.99
	[-282.22, 305.35]	[-342.12, 296.72]	[-158.33, 135.95]	[-261.66, 285.25]	[-315.25, 338.26]	[-365.37, 434.36]	[-259.0, 304.97]
5	-5.11	15.04	-2.56	4.05	-17.6	-11.0	6.6
	[-223.06, 212.85]	[-172.29, 202.37]	[-112.32, 107.2]	[-198.48, 206.57]	[-211.25, 176.04]	[-268.81, 246.82]	[-201.78, 214.99]
10	-2.61	-2.9	2.14	-4.42	5.04	-1.53	-6.56
	[-86.2, 80.98]	[-75.81, 70.01]	[-39.85, 44.13]	[-82.37, 73.52]	[-70.3, 80.37]	[-101.46, 98.41]	[-86.78, 73.65]
16	8.06	1.22	2.29	-0.86	1.08	-2.07	-3.15
	[-90.16, 106.28]	[-82.58, 85.01]	[-47.0, 51.59]	[-93.39, 91.68]	[-85.96, 88.12]	[-119.16, 115.01]	[-98.64, 92.34]

Note: The table summarizes the difference of the Group Average Treatment Effects (GATEs) and Average Treatment Effects (ATEs) for the policy feature number of prenatal visits in the birthweight study. 90 % confidence intervals are given below the point estimates in square brackets. The evaluation points are given in the leftmost column. Value labels are given in Table 14.

Table S7: GATEs for mean blood pressure in the RHC study

	GATE	GATE-ATE
0	0.08	0.03
	[-0.02, 0.19]	[-0.06, 0.13]
35	0.11	0.06
	[0.04, 0.17]	[0.01, 0.11]
38	0.1	0.05
	[0.05, 0.16]	[0.01, 0.1]
40	0.1	0.05
	[0.04, 0.15]	[0.01, 0.09]
41	0.1	0.05
	[0.04, 0.15]	[0.01, 0.09]
43	0.1	0.05
	[0.05, 0.14]	[0.01, 0.08]
45	0.09	0.05
	[0.05, 0.14]	[0.01, 0.08]
46	0.09	0.05
	[0.05, 0.14]	[0.01, 0.08]
47	0.09	0.05
	[0.05, 0.14]	[0.02, 0.08]
48	0.09	0.05
	[0.05, 0.14]	[0.02, 0.08]
49	0.1	0.05
	[0.05, 0.14]	[0.02, 0.08]
50	0.09	0.05
	[0.05, 0.14]	[0.02, 0.07]
51	0.09	0.04
	[0.05, 0.14]	[0.02, 0.07]
52	0.09	0.04
	[0.05, 0.14]	[0.02, 0.07]
53	0.09	0.04
	[0.05, 0.13]	[0.02, 0.07]
54	0.09	0.04
	[0.05, 0.13]	[0.01, 0.07]
55	0.09	0.04
	[0.04, 0.13]	[0.01, 0.07]
56	0.08	0.03
	[0.04, 0.13]	[0.01, 0.06]
57	0.08	0.03
	[0.04, 0.12]	[0.0, 0.06]
58	0.07	0.03
	[0.03, 0.12]	[-0.0, 0.06]
59	0.07	0.02

Table S7 continued from previous page

	GATE	GATE-ATE
	[0.03, 0.11]	[-0.01, 0.05]
60	0.07	0.02
	[0.02, 0.11]	[-0.01, 0.05]
61	0.06	0.01
	[0.02, 0.11]	[-0.02, 0.05]
62	0.06	0.01
	[0.01, 0.11]	[-0.02, 0.04]
63	0.06	0.01
	[0.01, 0.1]	[-0.02, 0.04]
65	0.05	0.0
	[0.01, 0.1]	[-0.03, 0.04]
66	0.05	0.0
	[0.0, 0.1]	[-0.03, 0.04]
67	0.05	-0.0
	[-0.0, 0.09]	[-0.04, 0.03]
68	0.04	-0.0
	[-0.0, 0.09]	[-0.04, 0.03]
70	0.04	-0.01
	[-0.01, 0.09]	[-0.05, 0.03]
72	0.03	-0.01
	[-0.01, 0.08]	[-0.05, 0.02]
77	0.01	-0.04
	[-0.04, 0.06]	[-0.08, 0.01]
99	0.0	-0.05
	[-0.05, 0.06]	[-0.09, 0.0]
106	-0.01	-0.06
	[-0.06, 0.05]	[-0.1, -0.01]
110	-0.01	-0.06
	[-0.06, 0.04]	[-0.1, -0.02]
112	-0.01	-0.06
	[-0.06, 0.04]	[-0.1, -0.02]
113	-0.01	-0.06
	[-0.07, 0.04]	[-0.1, -0.02]
115	-0.01	-0.06
	[-0.07, 0.04]	[-0.1, -0.02]
118	-0.02	-0.06
	[-0.07, 0.04]	[-0.11, -0.02]
121	-0.01	-0.06
	[-0.07, 0.04]	[-0.11, -0.02]
124	-0.01	-0.06
	[-0.07, 0.04]	[-0.1, -0.01]
127	-0.01	-0.05

Table S7 continued from previous page

	GATE	GATE-ATE
	[-0.06, 0.05]	[-0.1, -0.01]
129	-0.0	-0.05
	[-0.06, 0.05]	[-0.1, -0.01]
132	-0.0	-0.05
	[-0.06, 0.05]	[-0.1, -0.0]
135	-0.0	-0.05
	[-0.06, 0.05]	[-0.1, -0.0]
140	-0.0	-0.05
	[-0.07, 0.06]	[-0.1, -0.0]
145	-0.01	-0.06
	[-0.07, 0.05]	[-0.11, -0.0]
154	-0.0	-0.05
	[-0.07, 0.06]	[-0.11, 0.0]
170	0.01	-0.04
	[-0.06, 0.08]	[-0.1, 0.03]

Note: The table summarizes the Group Average Treatment Effects (GATEs) and the difference of the GATEs and Average Treatment Effect (ATE) for the policy feature blood pressure in the RHC study. The evaluation points are given in the leftmost column. 90 % confidence intervals are given below the point estimates in square brackets.

Table S8: GATEs for APACHE III in the RHC study

	**GATE**	**GATE-ATE**
15	-0.0	-0.05
	[-0.07, 0.06]	[-0.11, 0.01]
21	-0.0	-0.05
	[-0.05, 0.05]	[-0.09, -0.01]
24	0.01	-0.04
	[-0.04, 0.05]	[-0.08, -0.0]
27	0.01	-0.04
	[-0.04, 0.05]	[-0.08, -0.0]
29	0.0	-0.04
	[-0.04, 0.05]	[-0.08, -0.01]
31	0.0	-0.05
	[-0.04, 0.04]	[-0.08, -0.01]
33	-0.01	-0.05
	[-0.05, 0.04]	[-0.09, -0.02]
35	-0.01	-0.06
	[-0.05, 0.03]	[-0.09, -0.02]
36	-0.01	-0.06
	[-0.05, 0.03]	[-0.09, -0.03]
38	-0.01	-0.06
	[-0.05, 0.03]	[-0.09, -0.03]
39	-0.01	-0.06
	[-0.05, 0.03]	[-0.09, -0.03]
41	-0.01	-0.05
	[-0.05, 0.03]	[-0.08, -0.03]
42	-0.0	-0.05
	[-0.04, 0.04]	[-0.08, -0.02]
43	0.0	-0.05
	[-0.04, 0.04]	[-0.07, -0.02]
45	0.01	-0.03
	[-0.03, 0.05]	[-0.06, -0.01]
46	0.02	-0.03
	[-0.02, 0.06]	[-0.05, 0.0]
47	0.03	-0.02
	[-0.01, 0.07]	[-0.05, 0.01]
48	0.03	-0.01
	[-0.01, 0.08]	[-0.04, 0.01]
49	0.04	-0.01
	[-0.01, 0.08]	[-0.04, 0.02]
50	0.04	-0.0
	[-0.0, 0.09]	[-0.03, 0.02]
51	0.05	0.0

Table S8 continued from previous page

	GATE	GATE-ATE
	[0.0, 0.09]	[-0.03, 0.03]
52	0.06	0.01
	[0.01, 0.1]	[-0.02, 0.04]
53	0.06	0.02
	[0.02, 0.11]	[-0.01, 0.04]
54	0.07	0.03
	[0.03, 0.12]	[-0.0, 0.05]
55	0.08	0.03
	[0.04, 0.13]	[0.0, 0.06]
56	0.08	0.04
	[0.04, 0.13]	[0.01, 0.07]
57	0.08	0.04
	[0.04, 0.13]	[0.01, 0.07]
58	0.08	0.04
	[0.04, 0.13]	[0.01, 0.07]
59	0.08	0.03
	[0.04, 0.13]	[0.0, 0.07]
60	0.08	0.03
	[0.03, 0.13]	[0.0, 0.07]
61	0.08	0.04
	[0.04, 0.13]	[0.0, 0.07]
62	0.09	0.04
	[0.04, 0.14]	[0.01, 0.08]
63	0.1	0.05
	[0.05, 0.15]	[0.02, 0.09]
64	0.11	0.06
	[0.06, 0.16]	[0.02, 0.1]
65	0.11	0.06
	[0.06, 0.16]	[0.02, 0.1]
66	0.11	0.06
	[0.06, 0.16]	[0.02, 0.09]
68	0.08	0.03
	[0.03, 0.13]	[-0.01, 0.07]
69	0.07	0.02
	[0.01, 0.12]	[-0.02, 0.06]
70	0.06	0.01
	[0.0, 0.12]	[-0.03, 0.06]
72	0.05	0.0
	[-0.01, 0.12]	[-0.05, 0.06]
73	0.05	0.01
	[-0.01, 0.12]	[-0.05, 0.06]
75	0.05	0.0

Table S8 continued from previous page

	GATE	GATE-ATE
	[-0.02, 0.12]	[-0.06, 0.06]
77	0.04	-0.01
	[-0.03, 0.11]	[-0.06, 0.05]
80	0.04	-0.0
	[-0.02, 0.11]	[-0.06, 0.06]
82	0.07	0.02
	[-0.01, 0.14]	[-0.05, 0.08]
85	0.1	0.06
	[0.03, 0.18]	[-0.01, 0.12]
89	0.1	0.05
	[0.02, 0.18]	[-0.02, 0.12]
96	0.12	0.07
	[0.03, 0.21]	[-0.01, 0.15]
106	0.12	0.07
	[0.01, 0.22]	[-0.04, 0.17]

Note: The table summarizes the Group Average Treatment Effects (GATEs) and the difference of the GATEs and Average Treatment Effect (ATE) for the policy feature aps1 in the RHC study. The evaluation points are given in the leftmost column. 90 % confidence intervals are given below the point estimates in square brackets.

Table S9: GATEs and GATEs-ATE for primary diseases in the RHC study

	**GATE**	**GATE-ATE**
0	0.04	-0.01
	[0.0, 0.08]	[-0.03, 0.01]
1	0.06	0.01
	[0.01, 0.12]	[-0.04, 0.07]
2	0.04	-0.0
	[-0.01, 0.1]	[-0.05, 0.04]
3	0.02	-0.03
	[-0.04, 0.07]	[-0.07, 0.01]
4	0.12	0.07
	[0.02, 0.22]	[-0.02, 0.17]
5	-0.02	-0.07
	[-0.1, 0.06]	[-0.13, -0.0]
6	0.01	-0.04
	[-0.06, 0.07]	[-0.09, 0.01]
7	0.05	0.01
	[-0.01, 0.12]	[-0.04, 0.06]
8	0.09	0.04
	[0.04, 0.14]	[-0.0, 0.08]

Note: The table summarizes the Group Average Treatment Effects (GATEs) and the difference of the GATEs and Average Treatment Effect (ATE) for the policy feature cat1 in the RHC study. The evaluation points are given in the leftmost column. 90 % confidence intervals are given below the point estimates in square brackets. Value labels are given in Table 15.

Table S10: GATEs for age in the WIA programs study

	2 vs. 1	3 vs. 1	4 vs. 1	3 vs. 2	4 vs. 2	4 vs. 3
18	255.56	1374.69	1356.46	1119.13	1100.9	-18.23
	[42.01, 469.1]	[1106.26, 1643.11]	[1000.22, 1712.7]	[882.7, 1355.56]	[768.1, 1433.71]	[-388.64, 352.19]
19	354.59	1495.45	1546.4	1140.86	1191.81	50.94
	[186.53, 522.65]	[1276.97, 1713.94]	[1228.78, 1864.01]	[937.92, 1343.8]	[884.68, 1498.93]	[-286.43, 388.32]
20	439.24	1739.32	1830.61	1300.08	1391.37	91.29
	[277.96, 600.52]	[1505.86, 1972.78]	[1464.61, 2196.62]	[1074.39, 1525.77]	[1030.28, 1752.47]	[-307.31, 489.9]
21	435.17	1946.72	1975.65	1511.55	1540.48	28.92
	[273.14, 597.19]	[1709.81, 2183.64]	[1637.7, 2313.6]	[1281.94, 1741.17]	[1207.61, 1873.35]	[-346.15, 404.0]
22	355.89	2015.27	1941.27	1659.37	1585.37	-74.0
	[176.62, 535.17]	[1749.67, 2280.87]	[1609.52, 2273.01]	[1407.31, 1911.44]	[1264.36, 1906.38]	[-450.1, 302.1]
23	343.76	2072.72	2052.32	1728.96	1708.56	-20.41
	[162.21, 525.3]	[1810.88, 2334.56]	[1690.36, 2414.27]	[1481.64, 1976.29]	[1356.96, 2060.15]	[-419.43, 378.62]
24	421.83	2203.89	2190.13	1782.06	1768.3	-13.76
	[239.07, 604.59]	[1932.5, 2475.28]	[1828.09, 2552.18]	[1524.89, 2039.24]	[1416.78, 2119.82]	[-418.5, 390.98]
25	504.17	2296.84	2208.15	1792.68	1703.98	-88.7
	[322.85, 685.49]	[2019.58, 2574.11]	[1862.18, 2554.12]	[1528.24, 2057.11]	[1368.2, 2039.75]	[-484.61, 307.21]
26	482.67	2299.31	2184.02	1816.65	1701.35	-115.3
	[292.96, 672.37]	[2022.21, 2576.42]	[1802.57, 2565.46]	[1561.53, 2071.76]	[1335.56, 2067.14]	[-533.15, 302.55]
27	433.78	2288.82	2362.63	1855.04	1928.85	73.81
	[226.11, 641.44]	[1993.92, 2583.71]	[1952.66, 2772.6]	[1588.13, 2121.95]	[1538.52, 2319.18]	[-369.13, 516.75]
28	388.14	2227.25	2406.66	1839.11	2018.52	179.41
	[168.92, 607.36]	[1925.53, 2528.96]	[1959.71, 2853.61]	[1564.83, 2113.38]	[1589.61, 2447.43]	[-296.96, 655.79]
29	384.6	2084.31	2277.11	1699.71	1892.51	192.8
	[156.8, 612.4]	[1777.04, 2391.59]	[1851.3, 2702.93]	[1419.46, 1979.97]	[1485.77, 2299.25]	[-263.23, 648.83]
30	416.24	2063.18	2188.23	1646.94	1771.99	125.05
	[192.37, 640.11]	[1752.09, 2374.26]	[1767.57, 2608.89]	[1355.7, 1938.17]	[1365.79, 2178.19]	[-335.01, 585.11]
31	507.82	2206.3	2199.18	1698.47	1691.36	-7.12
	[281.21, 734.44]	[1895.52, 2517.07]	[1782.32, 2616.04]	[1408.58, 1988.37]	[1289.82, 2092.89]	[-461.5, 447.26]
32	564.35	2201.7	2071.91	1637.35	1507.56	-129.79
	[328.2, 800.5]	[1868.22, 2535.18]	[1658.52, 2485.31]	[1326.8, 1947.9]	[1112.44, 1902.69]	[-589.75, 330.18]
33	524.15	2026.16	1968.36	1502.02	1444.21	-57.81
	[285.95, 762.35]	[1696.51, 2355.82]	[1543.44, 2393.27]	[1198.12, 1805.91]	[1038.95, 1849.47]	[-522.75, 407.13]
34	429.74	1911.5	2006.31	1481.76	1576.57	94.81

Table S10 continued from previous page

	2 vs. 1	3 vs. 1	4 vs. 1	3 vs. 2	4 vs. 2	4 vs. 3
	[179.66, 679.82]	[1574.32, 2248.68]	[1581.86, 2430.76]	[1181.97, 1781.55]	[1181.18, 1971.97]	[-360.7, 550.32]
36	249.25	1901.9	2052.65	1652.65	1803.4	150.75
	[34.76, 463.73]	[1567.69, 2236.11]	[1585.07, 2520.23]	[1340.93, 1964.38]	[1351.62, 2255.19]	[-368.68, 670.17]
37	212.4	1896.08	2096.08	1683.68	1883.68	200.0
	[-9.13, 433.93]	[1558.58, 2233.59]	[1581.35, 2610.81]	[1373.63, 1993.74]	[1386.51, 2380.85]	[-358.58, 758.57]
38	287.26	1759.38	2098.9	1472.12	1811.64	339.52
	[53.15, 521.38]	[1421.9, 2096.86]	[1586.1, 2611.7]	[1163.49, 1780.74]	[1317.35, 2305.92]	[-211.3, 890.34]
39	406.76	1623.76	2028.75	1217.0	1621.99	404.99
	[172.77, 640.76]	[1299.56, 1947.95]	[1533.95, 2523.55]	[925.64, 1508.35]	[1148.06, 2095.91]	[-119.37, 929.36]
40	539.46	1647.26	2029.92	1107.79	1490.46	382.67
	[314.39, 764.53]	[1341.8, 1952.72]	[1491.83, 2568.01]	[827.45, 1388.13]	[966.22, 2014.69]	[-180.78, 946.11]
41	573.43	1758.74	2119.29	1185.3	1545.86	360.56
	[343.63, 803.23]	[1394.26, 2123.21]	[1647.76, 2590.83]	[843.01, 1527.59]	[1091.25, 2000.47]	[-174.89, 896.0]
42	505.17	1758.43	2223.17	1253.26	1718.0	464.74
	[275.1, 735.24]	[1396.09, 2120.78]	[1768.44, 2677.9]	[909.38, 1597.15]	[1277.84, 2158.16]	[-56.9, 986.37]
43	422.74	1628.86	2158.43	1206.12	1735.69	529.57
	[188.77, 656.72]	[1261.93, 1995.79]	[1702.62, 2614.24]	[858.68, 1553.56]	[1295.42, 2175.96]	[6.38, 1052.77]
44	346.06	1413.78	1854.78	1067.72	1508.73	441.0
	[97.98, 594.13]	[1085.57, 1741.99]	[1381.82, 2327.74]	[767.06, 1368.39]	[1054.44, 1963.01]	[-61.55, 943.55]
45	307.95	1330.92	1602.93	1022.98	1294.98	272.01
	[57.13, 558.76]	[1007.23, 1654.61]	[1088.77, 2117.09]	[736.3, 1309.65]	[803.28, 1786.69]	[-260.57, 804.59]
46	302.56	1288.14	1553.85	985.58	1251.29	265.72
	[55.15, 549.97]	[963.79, 1612.48]	[1058.28, 2049.43]	[696.9, 1274.25]	[778.29, 1724.3]	[-251.7, 783.13]
47	384.35	1291.85	1740.62	907.49	1356.27	448.77
	[153.22, 615.48]	[983.84, 1599.85]	[1221.68, 2259.56]	[626.35, 1188.64]	[852.81, 1859.73]	[-94.29, 991.84]
48	363.87	1252.53	1765.19	888.66	1401.32	512.66
	[135.68, 592.06]	[942.46, 1562.6]	[1307.13, 2223.26]	[611.53, 1165.79]	[964.88, 1837.76]	[28.35, 996.97]
49	275.4	1172.92	1676.94	897.53	1401.54	504.02
	[41.44, 509.35]	[855.04, 1490.81]	[1215.23, 2138.66]	[616.56, 1178.5]	[964.43, 1838.66]	[16.79, 991.24]
50	230.44	1208.75	1662.83	978.31	1432.39	454.08
	[-10.72, 471.6]	[858.67, 1558.82]	[1161.33, 2164.33]	[665.71, 1290.9]	[956.3, 1908.48]	[-85.42, 993.58]
51	262.93	1304.0	1802.81	1041.07	1539.88	498.81
	[28.0, 497.86]	[942.54, 1665.46]	[1189.92, 2415.7]	[704.99, 1377.14]	[941.6, 2138.15]	[-159.51, 1157.14]
52	232.74	1226.37	1848.04	993.64	1615.3	621.66

Table S10 continued from previous page

	2 vs. 1	3 vs. 1	4 vs. 1	3 vs. 2	4 vs. 2	4 vs. 3
	[-13.75, 479.22]	[853.8, 1598.95]	[1198.24, 2497.84]	[641.25, 1346.03]	[976.86, 2253.74]	[-75.23, 1318.56]
53	166.27	1051.11	1834.54	884.84	1668.27	783.43
	[-77.68, 410.22]	[702.45, 1399.77]	[1145.23, 2523.85]	[564.43, 1205.26]	[992.81, 2343.73]	[63.5, 1503.36]
54	100.1	944.3	1822.99	844.2	1722.9	878.7
	[-164.35, 364.54]	[567.18, 1321.41]	[1156.72, 2489.27]	[505.74, 1182.66]	[1077.72, 2368.08]	[179.74, 1577.66]
55	12.57	815.0	1658.31	802.44	1645.75	843.31
	[-257.2, 282.33]	[430.91, 1199.1]	[986.15, 2330.47]	[472.05, 1132.82]	[1002.77, 2288.73]	[144.61, 1542.01]
56	-24.76	748.37	1553.2	773.13	1577.96	804.83
	[-285.44, 235.91]	[373.58, 1123.15]	[844.77, 2261.62]	[442.37, 1103.89]	[891.81, 2264.11]	[67.73, 1541.93]
57	29.11	826.14	1593.19	797.03	1564.08	767.05
	[-222.25, 280.47]	[465.37, 1186.91]	[973.76, 2212.61]	[475.22, 1118.84]	[966.5, 2161.65]	[115.84, 1418.25]
59	125.12	768.51	1850.2	643.39	1725.08	1081.69
	[-144.45, 394.7]	[379.0, 1158.03]	[1164.12, 2536.28]	[284.29, 1002.49]	[1055.79, 2394.36]	[355.75, 1807.62]
60	117.35	705.26	2109.08	587.91	1991.73	1403.82
	[-183.4, 418.09]	[270.95, 1139.57]	[1389.68, 2828.47]	[189.8, 986.03]	[1293.59, 2689.87]	[638.59, 2169.05]
63	43.08	697.07	1932.05	653.99	1888.96	1234.97
	[-317.43, 403.6]	[166.94, 1227.2]	[945.63, 2918.46]	[190.17, 1117.81]	[936.54, 2841.39]	[206.3, 2263.65]
67	164.91	703.4	1599.68	538.49	1434.76	896.27
	[-182.83, 512.66]	[161.66, 1245.14]	[475.41, 2723.95]	[63.66, 1013.32]	[341.16, 2528.36]	[-273.56, 2066.11]

Note: The table summarizes the Group Average Treatment Effects (GATEs) for the policy feature age in the WIA study. The evaluation points are given in the leftmost column. 90 % confidence intervals are given below the point estimates in square brackets.

Table S11: GATEs - ATE for age in the WIA programs study

	**2 vs. 1**	**3 vs. 1**	**4 vs. 1**	**3 vs. 2**	**4 vs. 2**	**4 vs. 3**
18	-79.53	-265.1	-600.34	-185.57	-520.81	-335.24
	[-301.36, 142.3]	[-544.77, 14.57]	[-988.89, -211.78]	[-434.37, 63.23]	[-887.77, -153.84]	[-739.8, 69.32]
19	19.51	-144.33	-410.4	-163.84	-429.9	-266.07
	[-157.88, 196.89]	[-375.99, 87.33]	[-755.02, -65.77]	[-378.21, 50.54]	[-763.16, -96.65]	[-631.11, 98.98]
20	104.15	99.54	-126.18	-4.62	-230.34	-225.72
	[-62.69, 271.0]	[-139.38, 338.45]	[-498.48, 246.12]	[-231.53, 222.29]	[-595.05, 134.38]	[-628.53, 177.09]
21	100.08	306.94	18.85	206.86	-81.23	-288.09
	[-61.1, 261.27]	[71.45, 542.44]	[-328.74, 366.44]	[-18.06, 431.77]	[-421.74, 259.28]	[-669.43, 93.26]
22	20.81	375.48	-15.53	354.68	-36.34	-391.01
	[-149.09, 190.71]	[125.74, 625.23]	[-358.14, 327.08]	[119.05, 590.3]	[-368.8, 296.12]	[-770.53, -11.49]
23	8.67	432.94	95.52	424.27	86.85	-337.42
	[-160.78, 178.12]	[183.4, 682.48]	[-256.12, 447.16]	[190.29, 658.24]	[-253.92, 427.62]	[-724.3, 49.47]
24	86.74	564.11	233.34	477.37	146.59	-330.77
	[-81.2, 254.69]	[316.31, 811.91]	[-109.94, 576.62]	[244.21, 710.52]	[-186.28, 479.46]	[-710.25, 48.7]
25	169.08	657.06	251.35	487.98	82.27	-405.71
	[5.2, 332.97]	[406.85, 907.27]	[-79.32, 582.02]	[251.13, 724.83]	[-238.41, 402.95]	[-777.98, -33.44]
26	147.58	659.53	227.22	511.95	79.64	-432.31
	[-21.54, 316.71]	[410.19, 908.87]	[-126.52, 580.96]	[283.76, 740.14]	[-259.53, 418.81]	[-817.8, -46.82]
27	98.69	649.04	405.84	550.34	307.14	-243.2
	[-86.22, 283.61]	[385.87, 912.2]	[27.01, 784.66]	[313.48, 787.2]	[-53.91, 668.19]	[-649.92, 163.52]
28	53.05	587.46	449.87	534.41	396.81	-137.6
	[-145.6, 251.71]	[307.83, 867.09]	[40.18, 859.55]	[282.44, 786.37]	[5.48, 788.14]	[-575.62, 300.43]
29	49.52	444.53	320.32	395.01	270.8	-124.21
	[-153.74, 252.77]	[165.66, 723.4]	[-69.47, 710.1]	[141.61, 648.42]	[-101.19, 642.79]	[-542.34, 293.92]
30	81.15	423.39	231.43	342.24	150.28	-191.96
	[-122.16, 284.47]	[143.16, 703.63]	[-149.56, 612.43]	[84.45, 600.03]	[-214.52, 515.08]	[-604.6, 220.68]
31	172.74	566.52	242.38	393.78	69.65	-324.13
	[-29.77, 375.25]	[284.23, 848.8]	[-143.39, 628.15]	[132.95, 654.61]	[-300.71, 440.0]	[-743.46, 95.2]
32	229.26	561.92	115.12	332.65	-114.15	-446.8
	[19.92, 438.6]	[261.5, 862.33]	[-258.49, 488.73]	[53.71, 611.6]	[-470.72, 242.43]	[-863.42, -30.18]
33	189.06	386.38	11.56	197.32	-177.5	-374.82
	[-28.38, 406.51]	[82.24, 690.52]	[-377.76, 400.87]	[-79.37, 474.01]	[-545.77, 190.77]	[-800.07, 50.43]
34	94.65	271.71	49.52	177.06	-45.14	-222.2

Table S11 continued from previous page

	2 vs. 1	3 vs. 1	4 vs. 1	3 vs. 2	4 vs. 2	4 vs. 3
	[-128.07, 317.37]	[-34.46, 577.89]	[-339.77, 438.8]	[-94.61, 448.74]	[-407.91, 317.64]	[-641.42, 197.02]
36	-85.84	262.12	95.85	347.96	181.69	-166.26
	[-274.99, 103.31]	[-39.54, 563.77]	[-325.0, 516.7]	[66.21, 629.7]	[-225.12, 588.51]	[-636.07, 303.54]
37	-122.69	256.3	139.29	378.99	261.97	-117.02
	[-319.87, 74.5]	[-54.28, 566.88]	[-319.49, 598.06]	[94.15, 663.82]	[-179.78, 703.72]	[-619.73, 385.7]
38	-47.82	119.6	142.1	167.42	189.93	22.51
	[-252.51, 156.87]	[-182.81, 422.0]	[-307.44, 591.65]	[-109.46, 444.3]	[-242.86, 622.72]	[-464.17, 509.19]
39	71.68	-16.02	71.96	-87.7	0.28	87.98
	[-135.42, 278.78]	[-307.84, 275.79]	[-366.63, 510.54]	[-349.03, 173.63]	[-418.64, 419.2]	[-378.67, 554.63]
40	204.38	7.47	73.13	-196.91	-131.25	65.65
	[9.26, 399.49]	[-265.73, 280.68]	[-399.64, 545.9]	[-448.28, 54.47]	[-591.75, 329.25]	[-432.97, 564.28]
41	238.35	118.95	162.5	-119.4	-75.85	43.55
	[32.77, 443.93]	[-208.12, 446.02]	[-253.63, 578.63]	[-423.84, 185.05]	[-474.44, 322.75]	[-429.31, 516.4]
42	170.08	118.65	266.38	-51.43	96.29	147.73
	[-26.82, 366.98]	[-201.15, 438.45]	[-132.14, 664.89]	[-356.01, 253.14]	[-290.11, 482.69]	[-313.58, 609.04]
43	87.66	-10.92	201.64	-98.58	113.98	212.56
	[-112.18, 287.49]	[-333.53, 311.68]	[-188.59, 591.87]	[-405.18, 208.02]	[-263.12, 491.09]	[-241.69, 666.81]
44	10.97	-226.0	-102.01	-236.97	-112.98	123.99
	[-202.95, 224.9]	[-516.78, 64.78]	[-505.36, 301.34]	[-504.06, 30.12]	[-499.61, 273.64]	[-309.91, 557.88]
45	-27.14	-308.86	-353.86	-281.72	-326.73	-45.0
	[-247.78, 193.5]	[-596.28, -21.44]	[-794.76, 87.03]	[-533.68, -29.76]	[-745.37, 91.92]	[-502.38, 412.37]
46	-32.52	-351.64	-402.94	-319.12	-370.42	-51.3
	[-242.11, 177.06]	[-635.84, -67.45]	[-819.18, 13.3]	[-574.3, -63.94]	[-767.41, 26.58]	[-492.25, 389.66]
47	49.27	-347.94	-216.18	-397.2	-265.44	131.76
	[-143.45, 241.98]	[-611.69, -84.19]	[-649.04, 216.68]	[-639.49, -154.92]	[-685.57, 154.68]	[-325.33, 588.85]
48	28.79	-387.25	-191.6	-416.04	-220.39	195.65
	[-171.6, 229.17]	[-664.95, -109.55]	[-583.91, 200.71]	[-662.42, -169.65]	[-591.2, 150.42]	[-222.04, 613.33]
49	-59.69	-466.86	-279.85	-407.17	-220.17	187.01
	[-267.28, 147.9]	[-746.41, -187.31]	[-674.58, 114.88]	[-648.46, -165.88]	[-588.79, 148.46]	[-226.44, 600.45]
50	-104.65	-431.04	-293.97	-326.39	-189.32	137.07
	[-319.29, 110.0]	[-750.43, -111.64]	[-723.06, 135.13]	[-608.97, -43.81]	[-591.76, 213.12]	[-329.73, 603.86]
51	-72.15	-335.79	-153.99	-263.63	-81.83	181.8
	[-270.99, 126.68]	[-651.13, -20.44]	[-629.16, 321.19]	[-559.34, 32.07]	[-544.21, 380.55]	[-341.36, 704.96]
52	-102.35	-413.41	-108.76	-311.06	-6.41	304.65

Table S11 continued from previous page

	2 vs. 1	3 vs. 1	4 vs. 1	3 vs. 2	4 vs. 2	4 vs. 3
	[-312.76, 108.06]	[-747.98, -78.84]	[-672.18, 454.66]	[-630.86, 8.74]	[-561.19, 548.37]	[-308.08, 917.38]
53	-168.82	-588.67	-122.26	-419.85	46.56	466.42
	[-374.41, 36.77]	[-884.23, -293.12]	[-687.32, 442.8]	[-693.07, -146.64]	[-507.14, 600.26]	[-126.6, 1059.44]
54	-234.99	-695.49	-133.8	-460.5	101.19	561.69
	[-459.73, -10.25]	[-1018.25, -372.73]	[-709.78, 442.18]	[-750.59, -170.41]	[-457.14, 659.51]	[-42.79, 1166.17]
55	-322.52	-824.78	-298.48	-502.26	24.04	526.3
	[-551.78, -93.25]	[-1153.97, -495.59]	[-870.56, 273.59]	[-784.47, -220.05]	[-522.36, 570.43]	[-68.98, 1121.57]
56	-359.85	-891.42	-403.6	-531.57	-43.75	487.82
	[-583.13, -136.57]	[-1214.28, -568.55]	[-1012.22, 205.02]	[-816.17, -246.96]	[-632.97, 545.46]	[-145.87, 1121.51]
57	-305.98	-813.64	-363.61	-507.67	-57.63	450.03
	[-520.23, -91.72]	[-1127.86, -499.43]	[-890.67, 163.45]	[-789.65, -225.68]	[-566.14, 450.87]	[-108.0, 1008.07]
59	-209.96	-871.27	-106.6	-661.31	103.37	764.67
	[-443.99, 24.07]	[-1209.01, -533.53]	[-706.84, 493.65]	[-974.81, -347.81]	[-483.58, 690.32]	[129.21, 1400.13]
60	-217.74	-934.52	152.28	-716.78	370.02	1086.8
	[-480.86, 45.39]	[-1320.52, -548.53]	[-507.87, 812.43]	[-1072.04, -361.52]	[-272.64, 1012.68]	[384.83, 1788.78]
63	-292.0	-942.71	-24.75	-650.71	267.25	917.96
	[-614.88, 30.87]	[-1432.64, -452.78]	[-922.34, 872.84]	[-1082.24, -219.18]	[-599.84, 1134.35]	[-24.19, 1860.11]
67	-170.17	-936.38	-357.12	-766.21	-186.95	579.26
	[-485.84, 145.5]	[-1433.66, -439.1]	[-1405.27, 691.03]	[-1203.49, -328.93]	[-1208.0, 834.1]	[-511.7, 1670.22]

Note: The table summarizes the difference of the Group Average Treatment Effects (GATEs) and Averge Treatment Effects (ATEs) for the policy feature age in the WIA study. The evaluation points are given in the leftmost column. 90 % confidence intervals are given below the point estimates in square brackets.

Table S12: GATEs for claim to unemployment compensation in the WIA programs study

	2 vs. 1	3 vs. 1	4 vs. 1	3 vs. 2	4 vs. 2	4 vs. 3
0	312.52	1708.93	1934.92	1396.41	1622.4	225.99
	[214.03, 411.01]	[1566.86, 1851.0]	[1735.93, 2133.92]	[1264.4, 1528.42]	[1430.46, 1814.34]	[8.45, 443.54]
1	442.48	1310.68	2060.89	868.2	1618.41	750.21
	[251.63, 633.32]	[1084.98, 1536.37]	[1655.96, 2465.82]	[663.28, 1073.12]	[1224.68, 2012.15]	[338.46, 1161.97]

Note: The table summarizes the Group Average Treatment Effects (GATEs) for the policy feature unemployment compensation status in the WIA study. The evaluation points are given in the leftmost column. 90 % confidence intervals are given below the point estimates in square brackets. Value labels are given in Table 16.

Table S13: GATEs - ATE for claim to unemployment compensation in the WIA study

	**2 vs. 1**	**3 vs. 1**	**4 vs. 1**	**3 vs. 2**	**4 vs. 2**	**4 vs. 3**
0	-22.56	69.15	-21.87	91.71	0.69	-91.02
	[-50.13, 5.0]	[36.57, 101.72]	[-81.96, 38.22]	[62.07, 121.35]	[-57.86, 59.24]	[-152.09, -29.95]
1	107.39	-329.11	104.1	-436.5	-3.3	433.2
	[-23.81, 238.59]	[-484.17, -174.04]	[-181.94, 390.13]	[-577.57, -295.43]	[-281.99, 275.4]	[142.51, 723.9]

Note: The table summarizes the difference of Group Average Treatment Effects (GATEs) and Average Treatment Effects (ATEs) for the policy feature unemployment compensation status in the WIA study. The evaluation points are given in the leftmost column. 90 % confidence intervals are given below the point estimates in square brackets. Value labels are given in Table 16.

Table S14: Codebook for the maternal smoking during pregnancy study

variable	description
adequacy	adequacy of care (Kessner Index); 4 categories: adequate (1), intermediate (2), inadequate (3), unknown (4)
alcohol	indicator for alcohol consumption during pregnancy
cntyfipb	county of residence; 13 categories: Allegheny (3), Berks (11), Bucks (17), Chester (29), Delaware (45), Erie (49), Lancaster (71), Lehigh (77), Luzerne (79), Montgomery (91), Philadelphia (101), Westmoreland (129), York (133)
d	treatment variable; 6 categories: no smoking (0), 1-5 cigarettes per day (1), 6-10 cigarettes per day (2), 11-15 cigarettes per day (3), 16-20 cigarettes per day (4), more than 21 cigarettes per day (5)
dbirwt	outcome variable measuring birthweight in grams
deadkids_cat	number of dead kids; 6 categories: missing (-99), 1 dead child (1), 2 dead children (2), 3 dead children (3), 4 dead children (4), 5 or more dead children (5)
dfage_cat	age of father; 17 categories: missing (-99), younger than 15 years (0), 15-19 years (15), 20-24 years (20), 25-29 years (25), 30-34 years (30), 35-39 years (35), 40-44 years (40), 45-49 years (45), 50-54 years (50), 55-59 years (55), 60-64 years (60), 65-69 years (65), 70-74 years (70), 75-79 years (75), 80-84 years (80), older than 85 years (85)
dfeduc	education of father; 19 categories: no formal education (0), number of years of elementary school (1)-(8), 1 year of high school (9), 2 years of high school (10), 3 years of high school (11), 4 years of high school (12), 1 year of college (13), 2 years of college (14), 3 years of college (15), 4 years of college (16), 5 or more years of college (17), (99) not stated
dlivord_cat	live order; 6 categories: (-99) missing, (1) first, (2) second, (3) third, (4) forth, (5) fifth or more
dmage_cat	age of mother; 8 categories: younger than 15 years (0), 15-19 years (1), 20-24 years (2), 25-29 years (3), 30-34 years (4), 35-39 years (5), 40-44 years (6), older than 45 years (7)
dmar	indicator for marital status of mother
dmeduc	education of mother; 19 categories: no formal education (0), number of years of elementary school (1)-(8), 1 year of high school (9), 2 years of high school (10), 3 years of high school (11), 4 years of high school (12), 1 year of college (13), 2 years of college (14), 3 years of college (15), 4 years of college (16), 5 or more years of college (17), (99) not stated
foreignb	indicator if mother was born abroad
frace	race of father; 4 categories: other (0), black (1), hispanic (2), white (3)
mrace	race of mother; 4 categories: other (0), black (1), hispanic (2), white (3)
nprevist_cat	total number of prenatal care visits grouped; 5 categories: missing (-99), 0-4 visits (0), 5-9 visits (5), 10-15 visits (10), 16 or more prenatal care visits (16)
trimester	trimester when prenatal care began; 3 categories: first trimester (1), second trimester (2), third trimester (3)
years_cat	number of years since last birth grouped; 6 categories: missing (-99), 1 year (1), 2 years (2), 3 years (3), 4 years (4), more than 5 years (5)

Table S15: Codebook for the RHC study

variable	description
adld3pc	index of activities of daily living 2 weeks prior to admission
age	age in years
alb1	albumin level in grams per deciliter
amihx	indicator for definite myocardial infarction
aps1	acute physiology and chronic health evaluation score (APACHE III)
bili1	bilirubin level in milligrams per deciliter
ca	cancer; 3 categories: metastatic cancer (Metastatic), no cancer (No), cancer (Yes)
card	indicator for cardiovascular diagnosis
cardiohx	indicator for acute myocardial infarction, peripheral vascular disease, severe, and very severe cardiovascular symptoms (NYHA-classes III and IV)
cat1	primary disease; 9 categories: acute respiratory failure (0), congestive heart failure (1), chronic obstructive pulmonary disease (2), cirrhosis (3), colon cancer metastatic to the liver (4), non-traumatic coma (5), non-small-cell cancer of the lung, stage III or IV (6), multiorgan system failure with malignancy (7), indicator for multiorgan system failure with sepsis (8)
cat2	secondary disease; 3 categories: missings or others (0), multiorgan system failure with malignancy (1), multiorgan system failure with sepsis(2)
cat2_miss	indicator for missings in cat2
chfhx	indicator for congestive heart failure
chrpulhx	indicator for chronic, severe, or very severe pulmonary disease
crea1	creatinine level in milligrams per deciliter
das2d3pc	Duke activity status index (DASI) two weeks before admission
dementhx	indicator for dementia, stroke or cerebral infarct, Parkinson's disease
dnr1	indicator for do not resuscitate status on the first day
dth30	outcome indicator for mortality within 6 months
edu	education in years
gastr	indicator for gastrointestinal diagnosis
gibledhx	indicator for upper gastrointestinal bleeding
hema	indicator for hematologic diagnosis
hema1	hematocrit levels in percent
hrt1	heart rate in beats per minute
immunhx	indicator for immunosuppression, organ transplant, HIV positivity, diabetes mellitus with or without end organ damage, connective tissue disease
income	income in US dollars; 4 categories: below 11k (0), 11-25k (1), 25-50k (2), above 50k (3)
liverhx	indicator for cirrhosis, hepatic failure
malighx	indicator for solid tumor, metastatic disease, chronic leukemia/myeloma, acute leukemia, lymphoma
meanbp1	mean blood pressure in millimeters of mercury
meta	indicator for metabolic diagnosis
neuro	indicator for neurological diagnosis
ninsclas	medical insurance; 6 categories: Medicaid (0), Medicare (1), Medicare and Medicaid (2), no medical insurance (3), private medical insurance (4), private medical insurance and Medicaid (5)

continued on next page

Table S15 – continued from previous page

**variable**	**description**
ortho	indicator for orthopedic diagnosis
paco21	partial pressure of arterial carbon dioxide in millimeters of mercury
pafi1	ratio between partial pressure of arterial oxygen in millimeters of mercury and fraction of inspired oxygen
ph1	potential of hydrogen (pH) at logarithmic scale
pot1	blood potassium level in millimoles per liter
psychhx	indicator for psychiatric history, active psychosis or severe depression
race	race; 3 categories: African American (0), other race (1), Caucasian (2)
renal	indicator for renal diagnosis
renalhx	indicator for chronic renal disease, chronic hemodialysis, or peritoneal dialysis
resp	indicator for respiratory diagnosis
resp1	respiratory rate
scoma1	Glasgow coma score
seps	indicator for sepsis diagnosis
sex	indicator for gender (1=male)
sod1	sodium level in milliequivalents per liter
surv2md1	probability of surviving two months (based on support model estimations)
swang1	treatment indicator for right heart catheterisation
temp1	body temperature in degrees Celsius
transhx	indicator for transfer exceeding 24 hours from another hospital
trauma	indicator for trauma diagnosis
urin1	urine output per day in milliliters
urin1_miss	indicator for missings in urin1
wblc1	white blood cells in thousands per cubic millimeter
wtkilo1	weight in kilograms

Table S16: Codebook for the WIA programs study

**variable**	**description**
age	age in years
asian	indicator for Asian
black	indicator for African American
disab	indicator for disability status
earnings	outcome: difference in average earnings between four quarters after exiting the program and three quarters before entering
educ_col	indicator for bachelor's degree
educ_nod	indicator for no high school
educ_sec	indicator for high school
emp_b1	indicator for employment one quarter before treatment
emp_b2	indicator for employment two quarters before treatment
emp_b3	indicator for employment three quarters before treatment
female	indicator for female
latino	indicator for Hispanic
lowinc	indicator for low income status
mtreat	four-level treatment: core service (1), intensive service (2), basic/general training service (3), occupational training service (4)
occup	occupation according to the Standard Occupational Classification of the U.S. Bureau of Labor Statistics; 24 categories: 0, 11, 13, 15, 17, 19, 21, 25, 27, 29, 31, 33, 35, 37, 39, 41, 43, 45, 47, 49, 51, 53, 55, 99, see `https://www.bls.gov/soc/socguide.htm`, where 0 stands for no occupation and 99 summarizes the occupations with less than 0.1% of the total number of observations
sec_b1	industry sector according to the North American Industry Classification System one quarter before treatment; 23 categories: 0, 11, 23, 31, 32, 33, 42, 44, 45, 48, 49, 51, 52, 53, 54, 56, 61, 62, 71, 72, 81, 92, 99, see `https://www.census.gov/naics/?58967?yearbck=2017` for a detailed description, where 0 stands for no sector and 99 summarizes the sectors with less than 0.1% of the total number of observations
sec_b2	industry sector two quarters before treatment; 23 categories identical to sec_b1
sec_b3	industry sector three quarters before treatment; 23 categories identical to sec_b1
uc	indicator for claims to unemployment compensation
unemp	indicator for unemployment
vet	indicator for veteran status
wage_b1	quarterly earnings in USD one quarter before treatment
wage_b2	quarterly earnings in USD two quarters before treatment
wage_b3	quarterly earnings in USD three quarters before treatment
white	indicator for Caucasian
